# Enhanced Fyn-tau and NR2B-PSD95 interactions in epileptic foci in experimental models and human epilepsy

**DOI:** 10.1093/braincomms/fcae327

**Published:** 2024-09-19

**Authors:** Marson Putra, Nikhil S Rao, Cara Gardner, Guanghao Liu, Jordan Trommater, Michael Bunney, Meghan Gage, Alexander G Bassuk, Marco Hefti, Gloria Lee, Thimmasettappa Thippeswamy

**Affiliations:** Department of Biomedical Sciences, College of Veterinary Medicine, Iowa State University, Ames, IA 50010, USA; Department of Biomedical Sciences, College of Veterinary Medicine, Iowa State University, Ames, IA 50010, USA; Department of Biomedical Sciences, College of Veterinary Medicine, Iowa State University, Ames, IA 50010, USA; Department of Internal Medicine, Carver College of Medicine, Carver College of Medicine University of Iowa, Iowa City, IA 52242, USA; Department of Biomedical Sciences, College of Veterinary Medicine, Iowa State University, Ames, IA 50010, USA; Department of Biomedical Sciences, College of Veterinary Medicine, Iowa State University, Ames, IA 50010, USA; Department of Biomedical Sciences, College of Veterinary Medicine, Iowa State University, Ames, IA 50010, USA; Department of Pediatrics, The University of Iowa Stead Family, Iowa City, IA 52242, USA; Department of Neurology, The University of Iowa Stead Family, Iowa City, IA 52242, USA; Iowa Neuroscience Institute (INI), College of Medicine, University of Iowa Carver, Iowa City, IA 52242, USA; Department of Pathology, Carver College of Medicine, University of Iowa, Iowa City, IA 52240, USA; Department of Internal Medicine, Carver College of Medicine, Carver College of Medicine University of Iowa, Iowa City, IA 52242, USA; Department of Biomedical Sciences, College of Veterinary Medicine, Iowa State University, Ames, IA 50010, USA

**Keywords:** Fyn, tau, seizure, pY18, neurodegeneration

## Abstract

Epilepsy and Alzheimer’s disease share some common pathologies such as neurodegeneration, seizures and impaired cognition. However, the molecular mechanisms of these changes are still largely unknown. Fyn, a Src-family non-receptor tyrosine kinase (SFK), and its interaction with tau in mediating brain pathology in epilepsy and Alzheimer’s disease can be a potential therapeutic target for disease modification. Although Fyn and tau pathology occurs in both Alzheimer’s disease and epilepsy, the dynamics of Fyn-tau and PSD95-NR2B interactions affected by seizures and their impact on brain pathology in epilepsy have not been investigated. In this study, we demonstrate a significant increase of Fyn-tau interactions following seizure induction by kainate in both acute and chronic rodent models and in human epilepsy. In the early phase of epileptogenesis, we show increased Fyn/tau/NR2B/PSD95/neuronal nitric oxide synthase complexes after status epilepticus and a postsynaptic increase of phosphorylated tau (pY18 and AT8), Fyn (pSFK-Y416), NMDAR (pNR2B-Y1472) and neuronal nitric oxide synthase. Hippocampal proximity ligation assay and co-immunoprecipitation revealed a sustained increase of Fyn-tau and NR2B-PSD95 complexes/binding in rat chronic epilepsy at 3 months post-status epilepticus. Enhanced Fyn-tau complexes strongly correlated with the frequency of spontaneously recurring convulsive seizures and epileptiform spikes in the chronic epilepsy model. In human epileptic brains, we also identified increased Fyn-tau and NR2B-PSD95 complexes, tau phosphorylation (pY18 and AT8) and Fyn activation (pSFK-Y416), implying the translational and therapeutic potential of these molecular interactions. In *tau* knockout mice and in rats treated with a Fyn/SFK inhibitor saracatinib, we found a significant reduction of phosphorylated Fyn, tau (AT8 in saracatinib-treated), NR2B and neuronal nitric oxide synthase and their interactions (Fyn-tau and NR2B-PSD95 in saracatinib-treated group; NR2B-PSD95 in *tau* knockout group). The reduction of Fyn-tau and NR2B-PSD95 interactions in the saracatinib-treated group, in contrast to the vehicle-treated group, correlated with the modification in seizure progression in the rat chronic epilepsy model. These findings from animal models and human epilepsy provide evidence for the role of Fyn-tau and NR2B-PSD95 interactions in seizure-induced brain pathology and suggest that blocking such interactions could modify the progression of epilepsy.

## Teaser

Putra *et al.* report that epilepsy and Alzheimer’s disease may share certain similar pathomechanisms involving Fyn and tau interactions. They highlight the role of Fyn-tau and NMDAR postsynaptic density protein-95 complexes in mediating hyperexcitability, seizure-induced neuropathology and spontaneous seizures. These interactions could be potential therapeutic targets for epilepsy and seizure-related disorders.

## Graphical Abstract

**Graphical Abstract:**
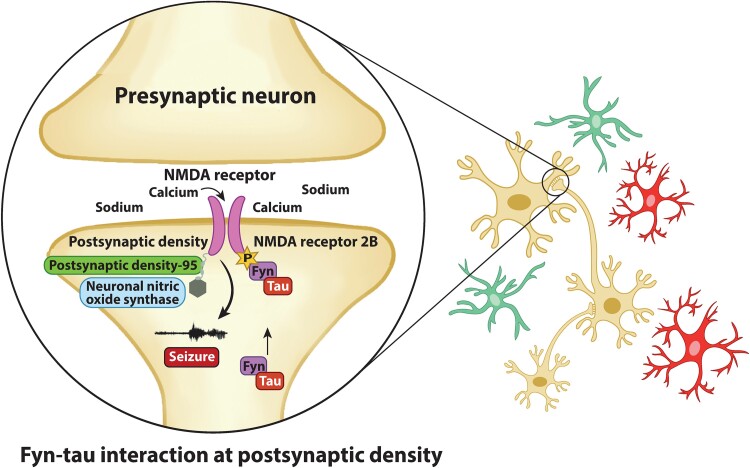

## Introduction

Epilepsy is characterized by the occurrence of spontaneously recurring seizures (SRS), affecting approximately 65 million people worldwide.^1^ One-third of epilepsy patients are refractory to anti-seizure drugs, prompting a quest for a better understanding of pathomechanisms of drug-resistant epilepsy. Aberrant network circuitry has been reported in patients with epilepsy and Alzheimer’s disease^2^ with an incidence of seizures in 10–20% and epilepsy in 6% of Alzheimer’s disease cases.^3,4^ The early Alzheimer’s disease–epilepsy prevalence is 87-fold higher than the general population.^5^ The comorbidity of Alzheimer’s disease and epilepsy and network dysfunction suggest that the two diseases may share some overlapping mechanisms. The role of tau or Fyn or both as shared mechanisms of neuronal hyperexcitability and seizures has been reported.^6-11^. Our study is the first to demonstrate Fyn-tau interactions in a pentylenetetrazole (PTZ) acute seizure mouse model.^11^

Tau is an axonal microtubule-associated protein which regulates microtubule stability and axonal transport of micromolecules under physiological condition.^12^ However, neurological disorders such as seizures can phosphorylate tau and facilitate interactions with other proteins to impair neuronal function.^13,14^ Seizures induced by the glutamate agonist kainate (KA) cause sustained increase in tau phosphorylation within 24 h,^14^ suggesting the role of tau pathology in seizure-mediated neurotoxicity. Hyperphosphorylation of tau has been reported in the brains of temporal lobe epilepsy (TLE) patients that correlated with generalized seizures^15^ and cognitive impairments.^15-17^ Moreover, tau promotes network hyperexcitability in tandem with Aβ in Alzheimer’s disease, and reducing tau decreases epileptiform spikes in mouse models of Alzheimer’s disease.^6,7^ Tau loss dampens neuronal excitability in the mouse and drosophila genetic models of epilepsy^8^ and in the mouse model of Dravet syndrome.^10^ Antisense reduction of tau in adult mice protected against seizures,^9^ indicating the role of tau in seizures.^18,19^

Fyn, a Src-family non-receptor tyrosine kinase (SFK), interacts with tau in neurons and mediate tau-dependent network hyperexcitability.^20,21^ Neuronal Fyn is involved in neuronal development and synaptic plasticity through its interaction with NMDAR.^22^ The activation of Fyn is reported to accelerate tau pathology^23^ and worsen cognitive impairment and synaptic loss in Alzheimer’s disease mice.^24,25^ Fyn overexpression exacerbated epileptiform discharges and decreased seizure threshold in Aβ-forming mice while tau ablation was protective.^7^ Genetically ablated *fyn* or pharmacological inhibition of Fyn/SFK prevented neuronal loss and decreased network hyperexcitability and seizure progression.^24,26,27^ Fyn’s pro-convulsive property is also mediated through microglia, independent of tau, and Fyn/SFK inhibition significantly decreased epileptogenesis.^27,28^ These findings suggest the role of Fyn and tau in promoting seizures and epilepsy.

Fyn and tau independently or through their interactions promote pathogenesis in neurological disorders^28-31^. The proline-rich domain of tau containing PxxP_5/6_ motifs binds with Src homology (SH3) domain-containing proteins such as Fyn leading to tau phosphorylation.^21^ Through this interaction, tau is tyrosine-phosphorylated by Fyn at Y18 (tyrosine 18)^20,21^ and causes NMDAR-mediated neurotoxicity.^32^ Moreover, Fyn-tau binding increases pathological Fyn activation.^33^ Importantly, tau and Fyn translocate to the postsynaptic density (PSD) where Fyn modulates synaptic plasticity through the phosphorylation of the NR2B subunit of NMDAR at Y1472 and stabilizes the coupling of NR2B with PSD protein 95 (PSD95) and promoting hyperexcitability and neurotoxicity.^34^

While Fyn-tau interaction is extensively studied in Alzheimer’s disease models, to our knowledge, its role in *status epilepticus* (SE) or epilepsy models remains poorly understood, despite the evidence of common brain pathology and dysfunctions in Alzheimer’s disease and epilepsy. Using an acute mouse and rat SE model and a rat chronic epilepsy model, we demonstrate increased Fyn-tau and NR2B-PSD95 interactions and their direct correlation with increased SRS frequency. We also validated their roles by disrupting their interactions in a *tau* knockout (KO) mouse model or by treating with a Fyn/SFK inhibitor, saracatinib (SAR) (AZD0530), in the rat chronic epilepsy model. A cohort of human brains from patients with epilepsy also revealed increased Fyn-tau and NR2B-PSD95 interactions. Therefore, Fyn-tau interaction may be a potential therapeutic target for seizures and seizure-induced neuropathology in epilepsy.

## Materials and methods

### Experimental animals and care

All animal procedures were performed as per the approved Institutional Animal Care and Use Committee of Iowa State University and ARRIVE (Animal Research: Reporting of In Vivo Experiments) guidelines. Adult mixed sex cohorts of mice were used in this study. The wild-type (WT) mice (7–8 weeks) were bred on C57BL/6J x S129 genetic background as previously described.^35^ The age-matched tau^−/−^ (KO) mice on C57BL/6J background were purchased from Jackson Laboratory (USA) and maintained in our laboratories. Sprague Dawley rats (7–8 weeks) were purchased from Charles River Laboratories (USA). All animals were housed in controlled environments on a 12 h light/dark cycle at 22^°^C with access to food and water *ad libitum*. At the end of each experiment, all animals were euthanized with pentobarbital sodium [100 mg/kg, intraperitoneal (i.p.); Hospira Inc., IL, USA].

### Human samples and ethic statement

All human tissues were collected and consented for research use under approved protocols by the Institutional Review Board (IRB) of the University of Iowa and the 1964 Helsinki Declaration and its later amendments. All samples were de-identified prior to use in accordance with the Human Research Protection IRB of the University of Iowa. Surgically resected temporal lobe samples were from patients with refractory epilepsy (*n*  *=* 6; five males and one female; mean age at the surgery = 38.1 years). Control brains were resected post-mortem with no history of known neurologic or psychiatric disorders (*n*  *=* 5; two males and one females; mean age at death = 35 years old). A post-mortem Alzheimer’s disease brain sample was used as a positive control for phosphorylated tau [AT8 (Serine 202/Threonine 205) and Y18] staining. The brain tissues were formalin-fixed, paraffin-embedded and sectioned for immunohistochemistry (IHC). Some tissues rapidly frozen were used for representative co-immunoprecipitation (Co-IP). Detailed information is available in Supplementary Table 1.

### Seizure and epilepsy induction

SE was induced by i.p. administration of the repeated low doses (RLD) of KA as reported previously.^36,37^ The animals received 5 mg/kg of KA at 30 min intervals and maxed at a total of 25 mg/kg. All experimental groups were blinded. KA injection was discontinued once the animals reached continuous convulsive stages (Stages 3–5 based on Racine scale^38^). After administration of KA, behavioural seizures were scored to determine SE severity as previously described.^37^ Two hours after the onset of first convulsive seizures, SE was terminated with diazepam (DZP, 5 mg/kg, i.p.; Hospira Inc., IL, USA). Age-matched controls were given an equal volume and number of injections of sterile water; however, DZP was not given to the control group. Animals were terminated at 24 h post-SE for acute study or at 3 months post-SE in the chronic study.

### EEG implantation for chronic seizure monitoring

Rats were individually housed in a standard laboratory cage and placed on the PhysioTel receiver pad [Data Science International (DSI), MN, USA] throughout the recording period. The rats were housed in a minimum electrical noise and soundproof environment for EEG acquisition. The details of EEG electrode implantation for surgical procedures are described in our previous publications.^27,39^ Briefly, a cohort of rats was anaesthetized, and a radio transmitter, CTA-F40 (DSI, MN, USA), was implanted subcutaneously, and the bipolar electrodes were placed on the parietal cortex epidurally. The electrodes were secured with dental cement, and the incision was closed. An antibiotic Baytril (5 mg/kg/SC/24 h; Bayer, USA) and fluids (s.c.) were given for 3 days. The EEG was continuously acquired (24/7) with Dataquest ART 4.3.2 (DSI) starting 72 h before KA challenge. Collected data were analysed using the NeuroScore 3.2.0 (DSI, MN, USA) software as described previously.^37^ The EEG recordings were transformed into time and frequency domains using a multitaper spectrogram.^40^

### Tau knockout mouse study and saracatinib treatment in the rat acute seizure and chronic epilepsy models

In mouse study, randomized WT and tau KO mice were challenged with RLD of KA to induce SE and euthanized 24 h post-SE for biochemical assays. In the rat acute study, after inducing SE, the rats were randomized and equally assigned between vehicle (VEH) and SAR (AZD0530) groups based on the matched SE scores/severity. Following 2 h post-DZP, the rats were dosed with SAR at 25 mg/kg/oral (PO)/12 h (two doses) or equivalent volume of VEH (0.5% hydroxypropyl methylcellulose and 0.1% Tween 80 and terminated 24 h post-SE). In the rat chronic epilepsy study for telemetry, SAR (20 mg/kg) or VEH dosing was started after the occurrence of first/second SRS post-SE for 14 days (14 doses, single dose/day). The rats were continuously EEG monitored for further 5 weeks after the last dose of SAR and euthanized thereafter.

### Tissue processing and immunolabelling

Tissue processing and immunolabelling of brain sections were done as previously described.^11^ Briefly, following euthanasia, animals were transcardially perfused with phosphate buffered saline (PBS) and/or 4% paraformaldehyde and processed for IHC as described previously.^41^ Dissected brain tissues were snap-frozen in liquid nitrogen and used for fractionation and western blotting.

### *In situ* proximity ligation assay

Proximity ligation assay (PLA) was performed as previously described.^11^ Briefly, after the brain sections were processed for antigen retrieval and blocked with donkey serum, the sections were incubated with primary antibodies, Fyn and tau (DA9), or NR2B and PSD95 antibodies to detect their interactions. MAP2 double staining was performed to visualize the localization of complexes within soma/dendrites. A Duolink *In Situ* Kit (Sigma-Aldrich, MO, USA) was used for PLA. The sections without primary antibodies served as negative controls for PLA experiments (Supplementary Fig. 5). Reagents and antibodies are listed in Supplementary Table 2.

### Tissue imaging and analysis

For immunostained brain sections imaging, Leica DMi8 (Leica, USA) or Keyence BZ-0023 (Keyence, Osaka, Japan) was used with a 20×/0.8 air-objective lens for analysis and 40×/1.3 and 4×/0.1 air-objective lens for illustration purpose. Additional details are included in the Supplementary Methods. The staining intensity expressed as integrated optical density was measured using ImageJ 2.0.0-rc-49/1.51d (NIH, USA) from the images taken with same exposure time for all. Cell counting was performed with CellProfiler Ver 4.2.1 (Broad Institute, MA, USA) with a uniform threshold adjusted for each cell type for all groups.

For PLA, the images were captured in 20 z-stacks, 1 µm intervals, with Leica DMi8 or Keyence BZ-0023 with a 40 × 1.3 air-objective lens. The images were then compressed into maximum intensity projection with LAS X software (Leica, Germany) for maximum visibility of puncta. For mouse/rat brain sections, up to 30 non-overlapping, z-stacked images of the hippocampus were obtained, and up to 40 images were collected from human brain sections. All images were taken with the same exposure time across groups and processed for unbiased punctate count using ImageJ as previously described.^11,42^

### Synaptosomal and postsynaptic density sample preparation

Subcellular fractionation of hippocampal and cortical tissues were carried out with some modifications from previously described studies.^35,43,44^ The detailed protocol is included in the Supplementary Methods. The lysates were homogenized in ice-cold sucrose buffer and centrifuged at 1000 × *g* at 4°C for 10 min generating the supernatant (S1) containing total protein (TP) and the nuclear-enriched pellets (P1). TP supernatants were further centrifuged at 12 000 × *g* for 20 min at 4°C to obtain cytosolic supernatant (S2) and crude synaptosomal fraction enriched pellets (P2), which were resuspended in washing buffer and centrifuged at 12 000 × *g* for 20 min at 4°C for two cycles. The supernatants were discarded, and the pellets were resuspended in buffer centrifuged at 12 000 × *g* for 20 min at 4°C. The supernatant (S3) of this process had soluble non-PSD fractions rich in extrasynaptic proteins and the pellet (P3) contained PSD fraction, which was resuspended in buffer and centrifuged at 10 000 × *g* for 15 min. The resultant supernatants (S4) contained detergent-insoluble PSD-enriched fraction. All fractions were aliquoted and stored at −80°C. The abundance of proteins was evaluated by western blotting with specific primary antibodies (Supplementary Fig. 5A and B).

### Immunoprecipitation

Briefly, the hippocampal tissues were lysed in Tris-NaCl-EDTA (TNE) buffer. Lysates were then centrifuged at 17 400*×g* for 35 min at 4°C. Protein concentrations were estimated using Bradford assay as previously described.^11^ An estimated 500 µg of lysates were incubated with 5 µg of tau-DA9 or PSD95 with agitation for 24 h at 4°C. Non-specific IgG isotype antibodies were also incubated with the samples used as the non-specific binding control (Supplementary Fig. 5D). The samples were later incubated with 25 µL of pre-washed A/G magnetic beads and rotated for 1 h at room temperature. The beads containing antigen-antibody complexes from the samples were separated from the rest by placing the tube on a magnetic stand (BioLegend Inc., CA, USA), and the flow-through samples were saved at −80°C. The beads were rinsed with TNE buffer three times followed by distilled water. The beads were then eluted in 30–40 µL of SDS-PAGE reducing sample buffer for 10 min at room temperature with mixing. Samples containing target proteins were then separated from the beads on a magnetic stand and loaded for western blotting. Details of reagents and chemicals used are available in Supplementary Table 2.

### Western blotting

The western blotting was carried out as previously described.^11^ Prepped samples (lysates or subcellular fractionation, immunoprecipitation) were resolved by 8 or 10% SDS-PAGE gels and transferred onto nitrocellulose membranes (Bio-Rad, USA). Subsequently, the membranes were rinsed with PBS, then blocked with Fluorescent-Blocking Buffer (Li-Cor, USA), incubated with primary followed by secondary antibodies, with wash in between, and visualized with LI-COR Odyssey 9120 IR imaging system (Li-Cor, USA). Quantitative densitometric analysis was performed using ImageJ 2.0.0-rc-49/1.51d (NIH). For Co-IP experiments, the levels of Fyn-tau complexes were expressed as relative to immunoprecipitated tau-DA9 protein levels while the levels of Fyn/tau/NR2B/PSD95 complexes were compared with total immunoprecipitated PSD95. Complete blot images are available in Supplementary Fig. 6.

### Enzyme linked immunosorbent assay

Phosphorylated tau (Ser202 and Thr205) levels in the sera from the rat chronic study were determined using enzyme link immunosorbent assay (ELISA) kits (LS Bios, GA, USA). The procedure followed was per the manufacturer’s instructions with minor modifications appropriate for serum samples. The detailed protocol is in Supplementary Methods. All assays were performed in triplicate.

### Study design and methodological rigour

The sample sizes were determined based on our pilot study and previous publications. Age-matched, randomized, mixed sex cohorts were used in mouse experiments, and SE severity was balanced between groups, whereas in rat experiments, only males were used since we did not observe sex interaction in the past and in our ongoing SAR experiments in a different project.^45^ Animals that died before the completion of the study were excluded from the analyses. All experiments and analyses were blinded until the data analyses were completed to minimize potential bias.

### Statistical analysis

Statistical analyses and graphical representation were performed and created using GraphPad Prism 9.0 and R-Studio version 1.1.463. Each marking on the bar graphs represents individual animal or human brain sample. Each data set was tested for normality with the Shapiro–Wilk test. An unpaired two-group comparison was analysed either with Student’s *t*-test for normally distributed data or Mann–Whitney for skewed data. Fisher’s exact test was used for a comparison of categorical variables. Multiple group comparison was analysed with one-way ANOVA or a repeated measure two-way ANOVA for two factor data with Tukey’s or Sidak’s *post hoc* when appropriate. Spearman’s correlation analysis was used to determine the relationship between parameters, fitted with the line of best fit at 95% confidence interval (CI). Multiple graphical data are presented as mean with error bars (± SEM, standard error mean). Statistical significance was determined if *P* ≤ 0.05. Details of statistical analyses are available in Supplementary Table 3.

## Results

### SE increases pTau (AT8, pY18), pFyn/SFK (Y416), pNR2B and fyn-tau and PSD95-NR2B/nNOS interactions at 24 h post-SE

**Figure 1:**
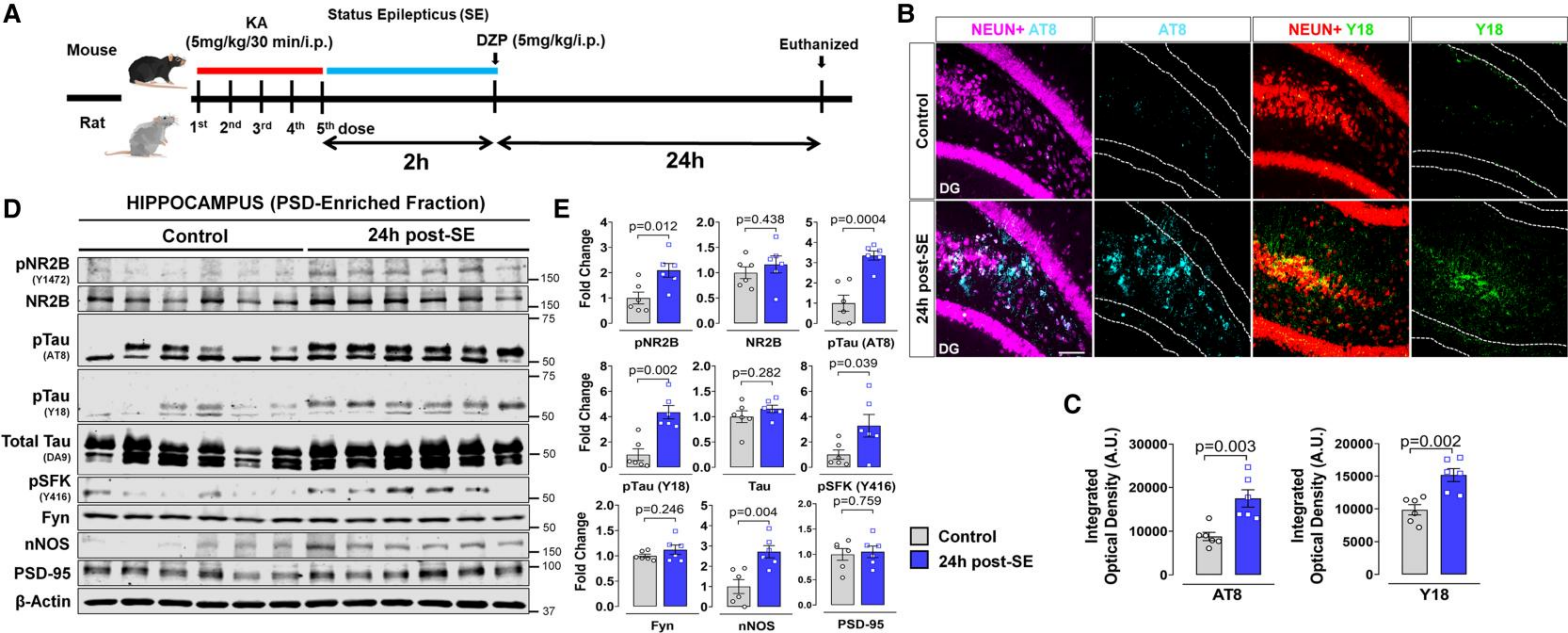
**SE increases pTau (AT8, Y18), pFyn/SFK (pSFK-Y416), pNR2B and Fyn-tau and NR2B/nNOS-PSD95 interactions at 24 h post-SE in KA animal models.** (**A**) Experimental design in a mouse and rat model of SE, induced by repeated low dose of KA. (**B**) Representative images of the dentate gyrus from AT8, Y18 and NeuN immunostained sections and their quantification [arbitrary unit (A.U.)] (**C**) from KA-treated and age-matched control mice. (**D**) Representative immunoblots of hippocampal PSD-enriched fraction from VEH- and KA-treated mice at 24 h post-SE revealed increased phosphorylation of NR2B (pNR2B), tau (AT8, Y18), Fyn (pSFK) and the levels of nNOS. The total Fyn, total Tau, NR2B and PSD-95 levels did not change post-KA. (**E**) Quantitative densitometry of the western blots. Uncropped blots are included in Supplementary Fig. 6. Scale bar 100 µm. Bar graphs displayed all data points and expressed as mean ± SEM. Dots represent individual animals. The Student’s *t*-test or the Mann–Whitney test was used for two-group comparison.

The experimental design is illustrated in Fig. 1A. At 24 h post-SE, there was a significant increase in hyperphosphorylated tau at AT8 (pSer202/Thr205) (*P* = 0.0027) and phospho-epitope Y18 (*P* = 0.0020) in the hippocampus of KA-treated mice (Fig. 1B and C). To determine whether the expression and activation of these proteins were altered at the PSD following SE, western blots of hippocampal PSD-enriched fractions were analysed. There was a significant upregulation of AT8 (*P* = 0.0004) and pY18 (*P* = 0.0022) in KA-treated mice at 24 h post-SE compared to controls with no change in the total tau levels (Fig. 1D and E). Although postsynaptic total Fyn levels were not altered, pSFK-Y416, a marker for Fyn/SFK activation, was significantly increased (*P* = 0.0398) in KA-treated mice compared to controls. Likewise, NR2B and PSD95 levels were unchanged (*P* = 0.4381, *P* = 0.7589) in the PSD fractions 24 h post-SE. However, relative to the VEH-treated group, phosphorylation of NR2B at Y1472 was significantly elevated (*P* = 0.0121) in KA-treated mice, indicating hyperactivation of NMDAR during early epileptogenesis (Fig. 1D and E). KA-treated mice also showed increased neuronal nitric oxide synthase (nNOS) (*P* = 0.0037) compared to VEH-treated animals (Fig. 1D and E). Similar changes occurred in the cortex of the KA-treated mice (Supplementary Fig. 1A, B).

**Figure 2:**
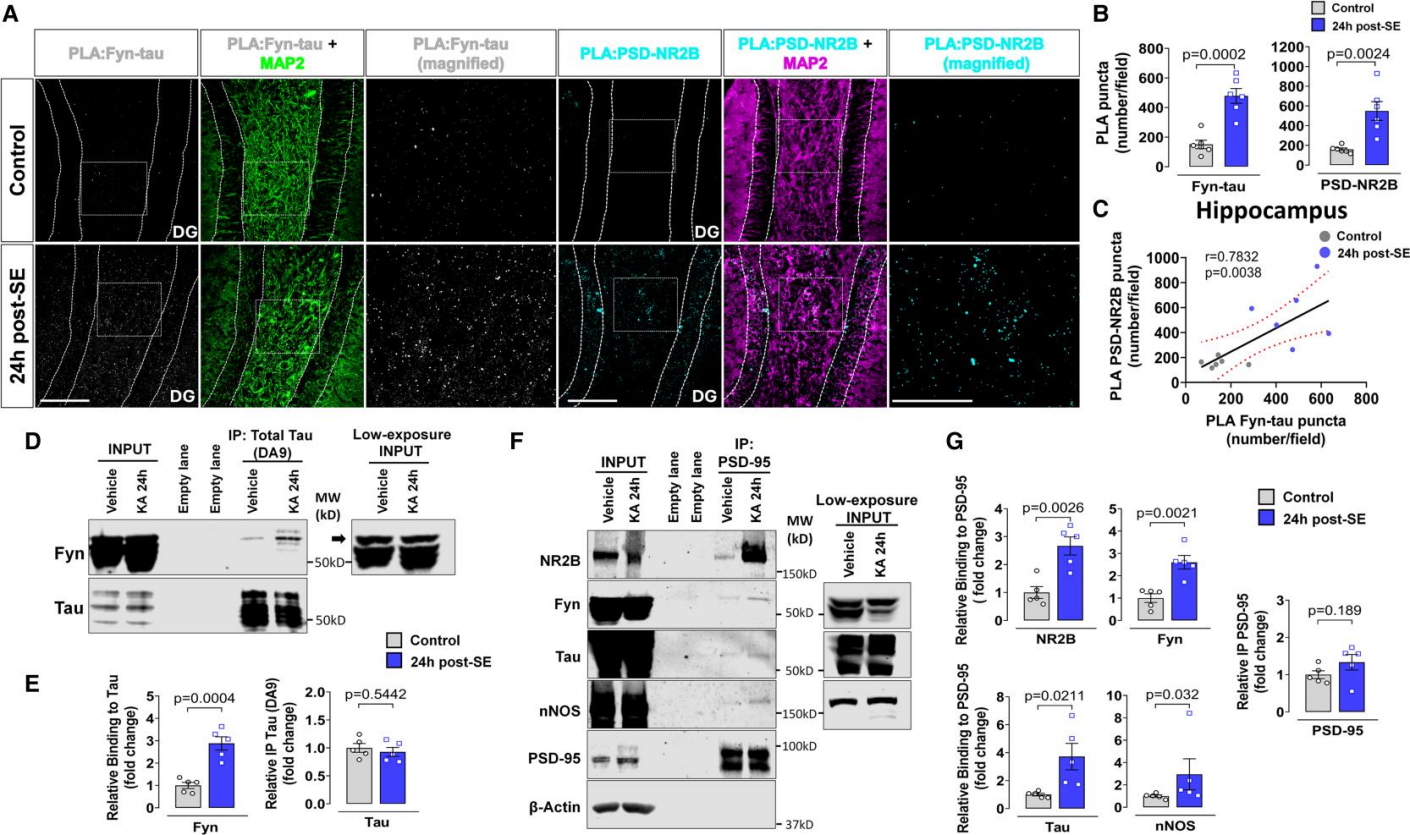
**SE increases Fyn-tau and NR2B/nNOS-PSD95 interactions at 24 h post-SE in KA animal models.** (**A**) Representative images of PLA for Fyn-tau (white dots) from DG and NR2B-PSD95 (cyan dots) co-labelled with a dendritic marker MAP2 (green/magenta) from CA3 hippocampus comparing VEH versus KA-treated mice at 24 h post-SE. (**B**) Quantification of PLA Fyn-tau and NR2B-PSD95 complexes. (**C**) Spearman correlation shows significant positive relationship between the Fyn-tau and NR2B-PSD95 complexes in the hippocampus. (**D**) Representative immunoprecipitation blots of the hippocampus confirming enhanced Fyn-tau binding in the KA-treated rats at 24 h post-SE versus VEH controls; low-exposure input blot showing the target bands (black arrow). (**E**) Quantification of immunoprecipitation blots. (**F**) Representatives immunoprecipitation blots of hippocampus show increased Fyn-tau, NR2B-PSD95 and nNOS-PSD95 complexes in rats treated with KA, but the TP levels (input) were unchanged; low-exposure input is also shown. (**G**) Quantification of immunoprecipitation blots. Uncropped blots are included in Supplementary Fig. 6. Scale bar 100 µm. Bar graphs displayed all data points and expressed as mean ± SEM. Dots represent individual animals. Two-group comparison used the Student’s *t*-test or the Mann–Whitney test. The red-dotted curvilinear in the correlational plot represents a 95% CI for the two means.

To further examine the extent of interactions between Fyn, tau, PSD95 and NR2B in response to SE induction, Fyn-tau and NR2B-PSD95 PLA complexes were quantified from the hippocampus (Fig. 2A and B). Relative to the VEH-treated group, the number of Fyn-tau (*P* = 0.0002) and NR2B-PSD95 (*P* = 0.0024) complexes was significantly higher in the hippocampus at 24 h post-SE (Fig. 2A and B). The Spearman correlation between Fyn-tau and NR2B-PSD95 PLA complexes revealed a significant (*P* = 0.0038) positive correlation (Fig. 2C). Furthermore, we examined the existence of such interactions in the rat SE model. The experimental design was similar to the mouse model (Fig. 1A). Hippocampal tissues from the rat model were used for Co-IP, which revealed that Fyn co-immunoprecipitated with tau significantly more (*P* = 0.0004) in KA-treated rats compared to the VEH group and the total Fyn and tau levels in both groups were unchanged (Fig. 2D and E). In addition, the levels of total tau precipitation using DA9 antibody also did not show differences (*P* = 0.5442) between groups (Fig. 2D and E). To examine whether SE also enhances NR2B-PSD95 complexes, PSD95 was immunoprecipitated and probed for co-immunoprecipitated NR2B. We observed a significant increase of NR2B Co-IP with PSD95 (*P* = 0.0026) in KA-treated group at 24 h post-SE compared to VEH-treated group (Fig. 2F and G). These findings from the 24 h post-SE in the rat model complemented the enhanced Fyn-tau and NR2B-PSD95 interactions revealed by increased PLA counts in the mouse SE model at 24 h post-SE (Fig. 2A and B). Further, PSD95-interacting proteins, Fyn (*P* = 0.0021), Tau (*P* = 0.0211) and nNOS (*P* = 0.0317), were also co-immunoprecipitated to a greater extent in the KA-treated groups at 24 h post-SE (Fig. 2F and G).

### Persistent increase in phosphorylated tau and Fyn/SFK in the rat chronic epilepsy model (3 months post-status epilepticus)

**Figure 3:**
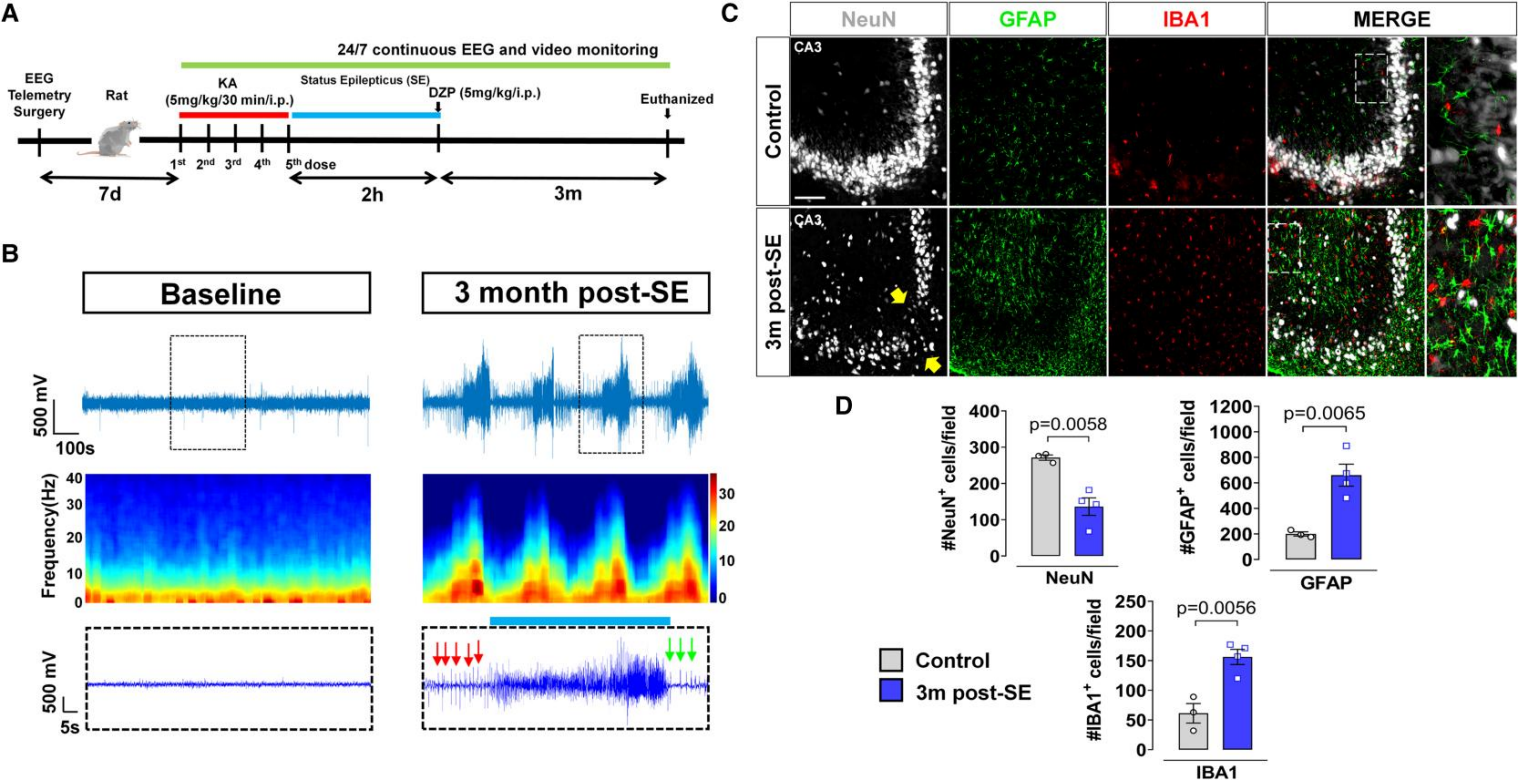
**Spontaneous recurrent seizures, gliosis and neuronal loss in the rat chronic epilepsy model (3 months post-SE).** (**A**) Experimental design. (**B**) Representative EEG traces and corresponding multi-tapered spectrograms comparing baseline and SRS demonstrating established epilepsy. Insets showing the baseline spikes and pre-ictal spikes (red arrows), a convulsive seizure (blue horizontal bar) and post-ictal spikes (green arrows). (**C**) Representative images of CA3 hippocampus immunolabelled with neuronal (NeuN), astroglial (GFAP) and microglial (IBA1) markers revealed astrogliosis and microgliosis with a significant neuronal loss (yellow arrows) in the KA group. Insets in dashed boxes show magnified images capturing the features of gliosis and neuronal loss in both groups. (**D**) Cell counts for NeuN, GFAP and IBA1. Scale bar 100 µm. Bar graphs displayed all data points and expressed as mean ± SEM. Dots represent individual animals. The Student’s *t*-test or the Mann–Whitney test was used for two-group comparison.

**Figure 4:**
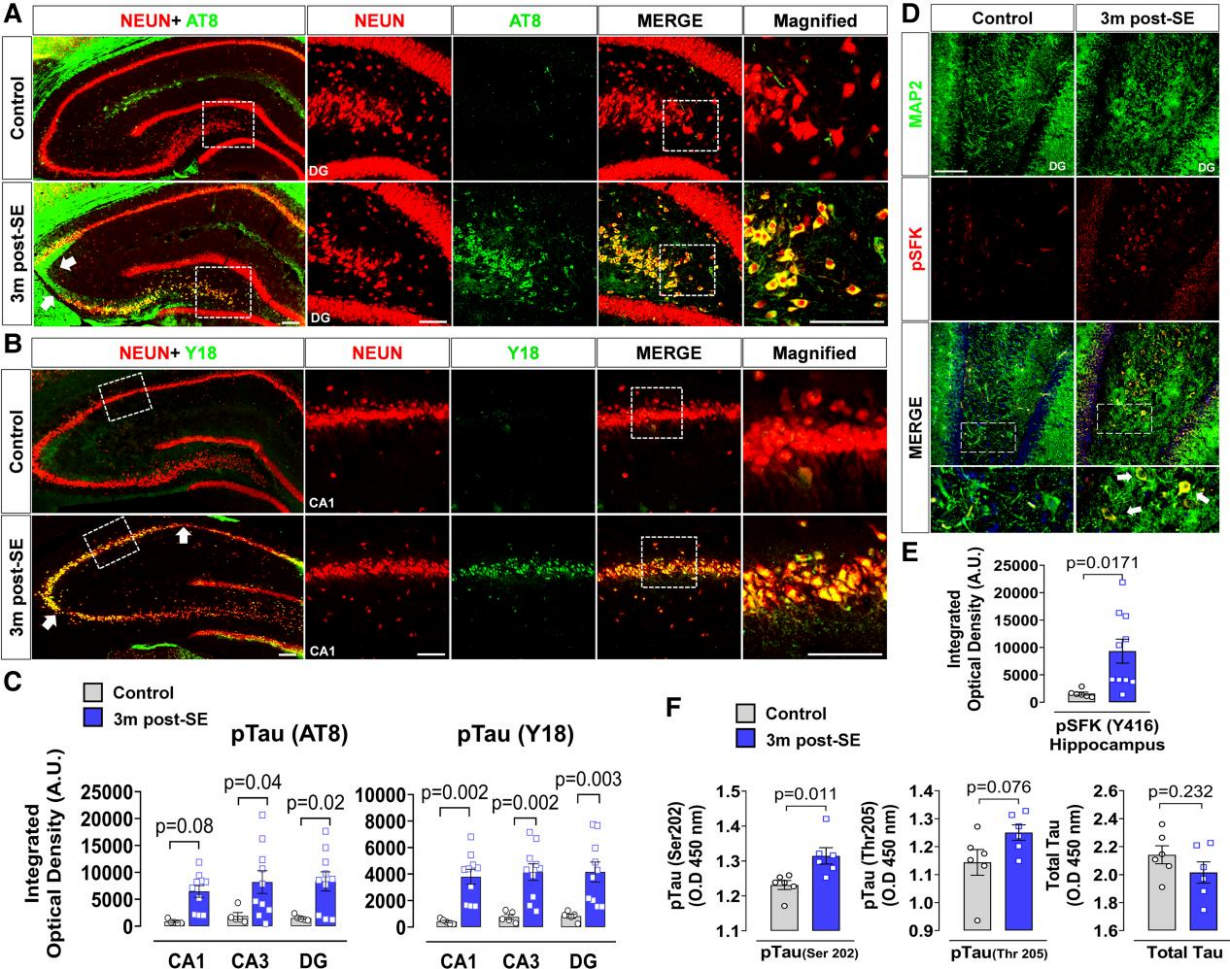
**Persistent increase in phosphorylated tau and Fyn in the rat chronic epilepsy model (3 months post-SE).** (**A, B**) Representative images of hippocampus immunolabelled with AT8 (**E**) and Y18 (**F**) colabelled with NeuN revealed the upregulation of AT8 and Y18 accompanied by neuronal loss (white arrows) in KA group. (**C**) Quantification of staining intensity of AT8 and Y18 in CA1, CA3 and DG [arbitrary unit (A.U.)]. Repeated measures two-way ANOVA with Sidak’s *post hoc*. (**D**) Representative immunostaining of pSFK (Y416) co-labelled with a dendritic marker MAP2 in DG showed elevated pSFK with predominant localization in the soma of neurons (white arrows) in the KA-treated rats. (**E**) Quantification of staining intensity of pSFK in the hippocampus. (**F**) ELISA for tau phosphorylation in serum of KA-treated rats at 3 months post-SE shows a significant increase of tau phospho-epitope Ser 202, but not Thr205 or total tau. Scale bar 100 µm. Bar graphs displayed all data points and expressed as mean ± SEM. Dots represent individual animals. The Student’s *t*-test or the Mann–Whitney test was used for two-group comparison.

The experimental design is illustrated in Fig. 3A. Continuous video-EEG monitoring for SRS confirmed the rats were epileptic (Fig. 3B). The brain IHC showed significant neuronal loss (NeuN, *P* = 0.0058), microgliosis (IBA1, *P* = 0.0056) and astrogliosis (GFAP, *P* = 0.0065) at 3 months post-SE relative to controls (Fig. 3C and D). The regions of neuronal loss in CA1 and CA3 are indicated by white arrows (Fig. 4A and B). The brain IHC also revealed significantly increased AT8 in CA3 and DG, but not CA1 of the hippocampus (*P* = 0.0133) in KA-treated rats compared to controls (Fig. 4A and C). We also found a significant increase of tau phosphorylation at tyrosine 18 (pY18) (*P* = 0.0019) (Fig. 4B and C), the primary target of Fyn kinase, and phosphorylated neuronal Fyn/SFK (pSFK-Y416) (*P* = 0.0171) in the hippocampus of KA group relative to controls (Fig. 4D and E). Interestingly, basal levels of Fyn and tau were relatively unchanged in the epileptic brain (Supplementary Fig. 2A and B). The serum phosphorylated tau at Ser202 (*P* = 0.0110), but not Thr205 (*P* = 0.0755) or total tau (*P* = 0.2322), was significantly increased in the epileptic rats (Fig. 4F).

### Enhanced Fyn-tau and NR2B-PSD95 interactions in rat epilepsy model: correlation with spontaneously recurring seizure and epileptiform spikes

**Figure 5:**
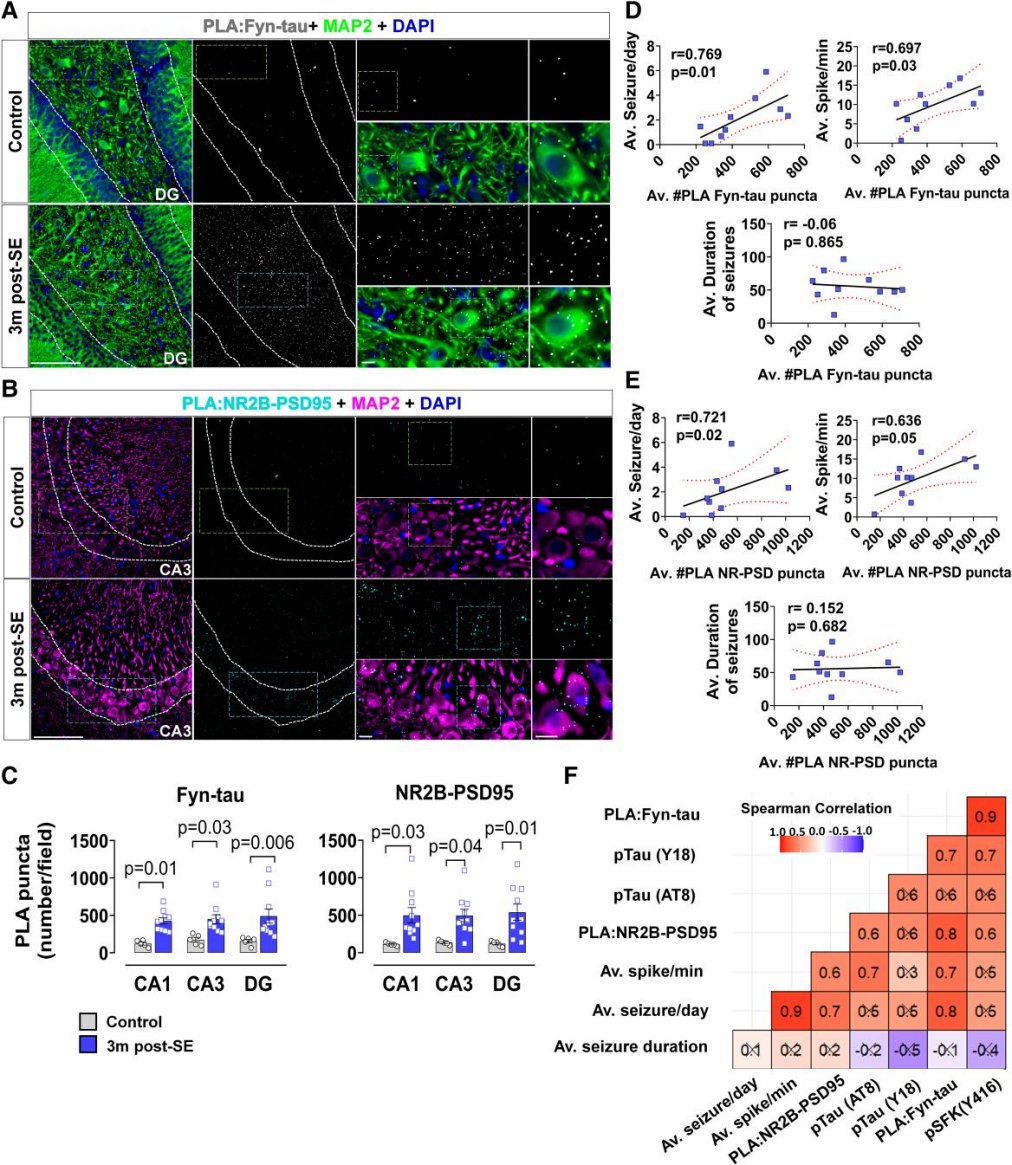
**Enhanced Fyn-tau and NR2B-PSD95 complexes in the rat chronic epilepsy: correlation with SRS and epileptiform spikes.** (**A–C**) Representative images of PLA for Fyn-tau interactions in DG (white dots like in **A**) and NR2B-PSD95 interactions in CA3 (cyan dots like in **B**) co-labelled with a dendritic marker MAP2 (green, in both **A** and **B**) show a significant increase of Fyn-tau/NR2B-PSD95 puncta/complexes (**C**) in KA-treated rats at 3 months post-SE. Repeated measures two-way ANOVA with Sidak’s *post hoc*. (**D, E**) Spearman correlation showing positive correlations between hippocampal Fyn-tau/NR2B-PSD95 complexes with the numbers of convulsive SRS [average (Av.) seizure/min] and epileptiform spike rate (Av. spike/min), but not with the duration of seizures (Av. duration of seizures). (**F**) Heatmap showing summary of Spearman correlation matrix between Fyn-tau associated proteins, interactions and EEG parameters. Black crosses correspond to non-significant correlations. Scale bar 100 µm. Bar graphs displayed all data points and expressed as mean ± SEM. Dots represent individual animals. The red-dotted curvilinear in the correlational plot represents a 95% CI for two means.

PLA analysis confirmed persistent enhancement of Fyn-tau (*P* = 0.0047) and NR2B-PSD95 (*P* = 0.0070) interactions in the hippocampus at 3-month post-SE (Fig. 5A–C). To determine whether these interactions are associated with seizure progression in KA-induced chronic epilepsy, we carried out a correlation analysis. We found that both Fyn-tau and NR2B-PSD95 PLA complexes in the hippocampus were positively correlated with the average number of seizures/day (Fyn-tau versus seizure/day, *r* = 0.769, *P* = 0.0126; NR2B-PSD versus seizure/day, *r* = 0.721, *P* = 0.0234) and the epileptiform spike rate (Fyn-tau versus spike/day, *r* = 0.697, *P* = 0.0306; NR2B-PSD versus spike/day, *r* = 0.636, *P* = 0.05) (Fig. 5D and E), suggesting that persistent increased Fyn-tau and NR2B-PSD95 interactions may have contributed to the occurrence of SRS and epileptiform spikes. However, there was no correlation between the number of Fyn-tau (r = −0.06) or NR2B-PSD95 (r = 0.152) PLA counts with the average SRS duration (Fig. 5D and E). A summary of the correlation matrix showed that the number of Fyn-tau PLA complexes appeared to correlate the most with other pathological parameters observed in the chronic study including the phosphorylated Fyn/SFK (pSFK, r = 0.9), Y18 (r = 0.7), and the number of NR2B-PSD95 PLA complexes (r = 0.8) (Fig. 5F).

### Increased pTau (AT8, Y18), pFyn/SFK and Fyn-tau and NR2B-PSD95 interactions in human epileptic brain

**Figure 6:**
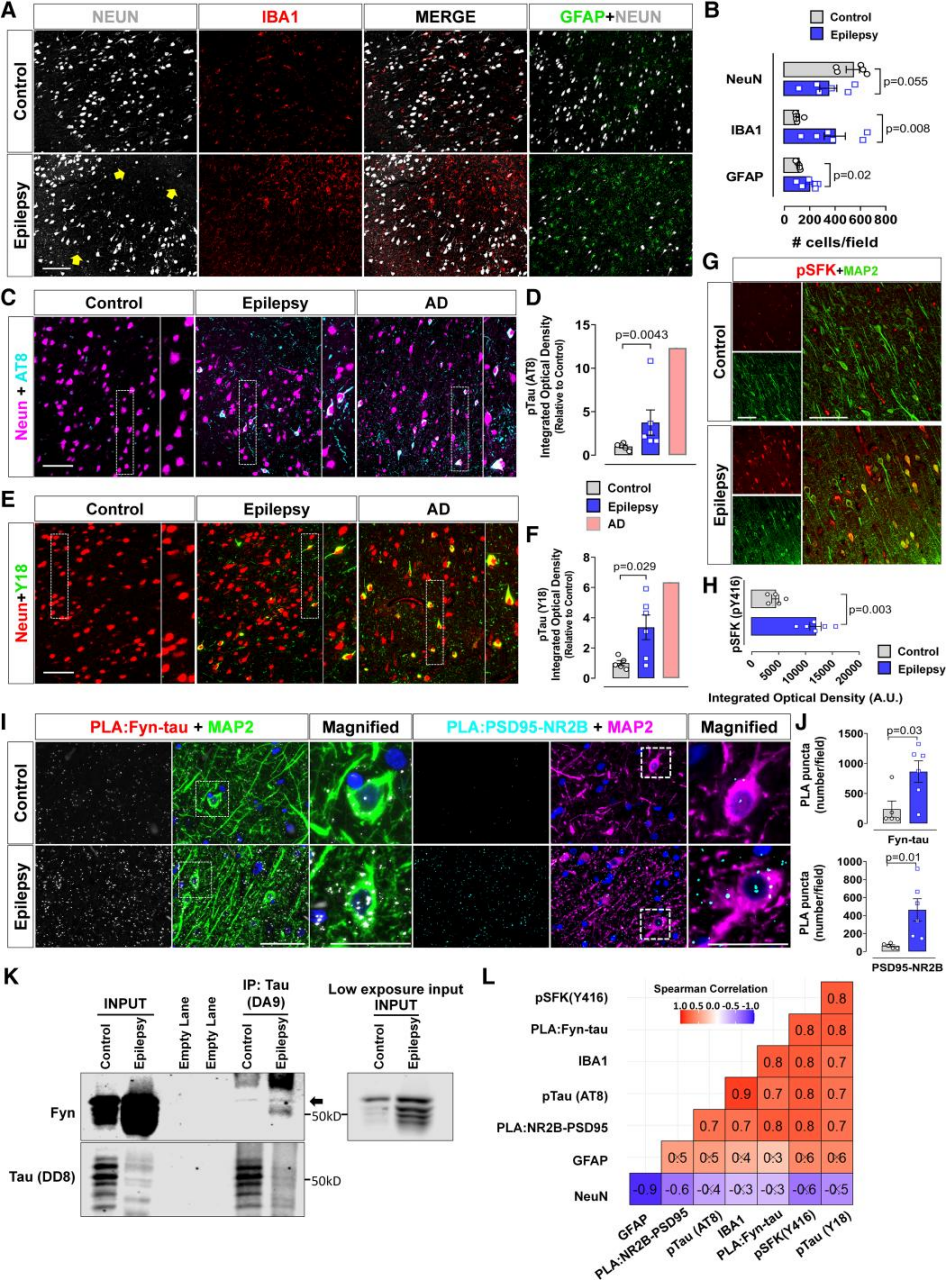
**Increased pTau (AT8, Y18), pFyn/SFK (Y416) and Fyn-tau and NR2B-PSD95 interactions in human epileptic brain.** (**A, B**) Representative images of the temporal cortex immunostained for NeuN, IBA1 and GFAP demonstrating microgliosis (red-labelled cells) and astrogliosis (green-labelled cells) with areas of reduced neurons (yellow arrows in the bottom panel) (**B**). (**C–F**) Representative images of AT8 (cyan in **C**), Y18 (green in **E**) and NeuN (magenta in **C** or red in **E**) showing upregulation of AT8 and Y18 in the epileptic and Alzheimer’s disease human brains (a positive control), compared to age-matched controls. Quantification of AT8 and Y18 staining intensity is shown in **D** and **F**. (**G, H**) Representative immunostaining of pSFK (Y416) (red in **G**) co-labelled with dendritic markers MAP2 (green in **G**) showing elevated pSFK in epileptic brain compared to age-matched controls (**H**) [arbitrary unit (A.U.)]. (**I, J**) Representative images of PLA for Fyn-tau interactions (white in **I)** and NR2B-PSD95 interactions (cyan in **I**) co-labelled with a dendritic marker MAP2 (green/magenta) showing increased interactions (puncta) for Fyn-tau/NR2B-PSD95 in epileptic versus control brains (**J**). (**K**) Representative Co-IP blots of human hippocampus showing relatively more binding of Fyn and tau in epilepsy than in control (black arrow); low-exposure input is also shown. Uncropped blots are included in Supplementary Fig. 6. (**L**) A heatmap showing summary of Spearman correlation matrix between Fyn-tau interactions and the other relevant signalling molecules. Black crosses correspond to non-significant correlations. Scale bar 100 µm. Bar graphs include all data points and expressed as mean ± SEM. Dots represent individual animals. Two-group comparison used the Student’s *t*-test or the Mann–Whitney test.

We further investigated whether similar brain pathology as in the rat model of chronic epilepsy and of increased Fyn-tau and NR2B-PSD95 interactions also occur in human patients with epilepsy. We found increased microgliosis (IBA1, *P* = 0.0087), astrogliosis (GFAP, *P* = 0.0226) and neuronal loss (NeuN, *P* = 0.0555), in the cortex of human patients with epilepsy relative to age-matched control brains (Fig. 6A and B). These observations suggest that in human epileptic brains, a similar neuropathology occurs as in the rat chronic model (Fig. 2C and D). We found that relative to age-matched control samples, there was a significant upregulation of AT8 (*P* = 0.0043) (Fig. 6C and D) and Y18 levels (*P* = 0.0292) in the epileptic brain (Fig. 6E and F). A positive control from an Alzheimer’s disease case confirmed the specificity of phospho-tau staining in the human epileptic brain (Fig. 6C–F). Activated Fyn/SFK, pSFK (Y416) was also significantly increased (*P* = 0.0003) in epileptic human brain relative to controls (Fig. 6G and H).

The PLA of the cortex of human epileptic brain for Fyn-tau (*P* = 0.0303) and NR2B-PSD95 (*P* = 0.0167) interactions revealed a significant upregulation compared to controls (Fig. 6I and J). In addition, Co-IP of cortical lysates confirmed increased binding of Fyn to tau, compared to age-matched control, further corroborating our PLA results (Fig. 6K). The Spearman correlation matrix revealed that Fyn-tau complexes positively correlated with the number of NR2B-PSD95 complexes (*r* = 0.79) (Fig. 6L). A positive correlation was also observed between the Fyn-tau PLA counts and Y18 expression (*r* = 0.83) (Fig. 6L). The number of PLA counts for Fyn and tau significantly correlated with all other parameters measured in this study, except for GFAP (*r* = 0.3) and NeuN (*r* = −0.3) counts.

### Tau knockout or Fyn/SFK inhibition mitigated status epilepticus–induced markers in the hippocampal postsynaptic density fraction 24 h post-status epilepticus

**Figure 7:**
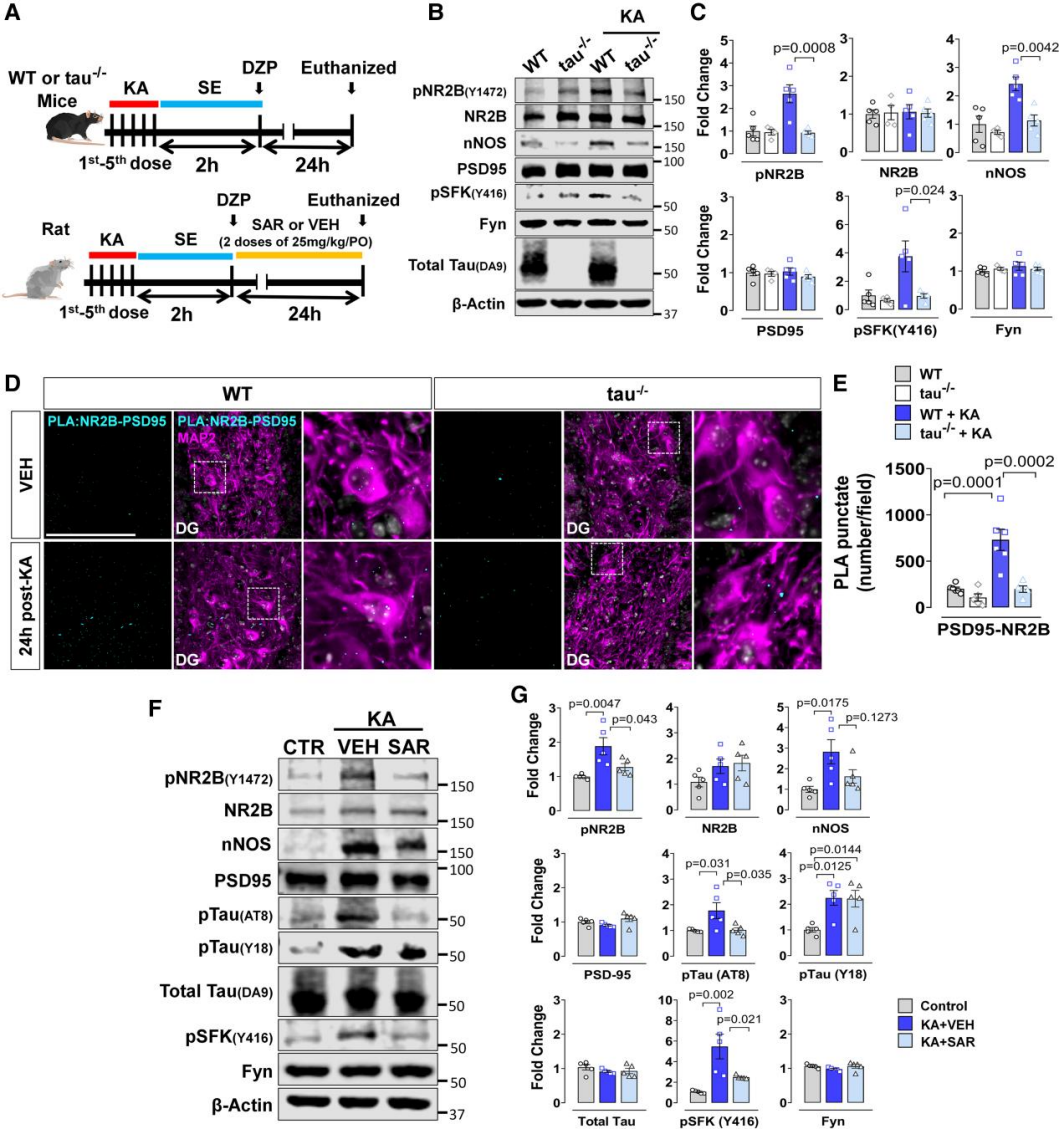
**Tau KO or Fyn/SFK inhibition mitigated SE-induced pathological markers in the hippocampal PSD fraction 24 h post-SE.** (**A**) Experimental design for repeated low dose of KA-induced seizure model in a mouse *tau* KO study and in rats with SAR or VEH treatment. (**B, C**) Representative immunoblots of hippocampal PSD-enriched fraction revealed the downregulation of pNR2B, pFyn/pSFK and nNOS levels in *tau* KO relative to WT control. The total Fyn, PSD95 and NR2B levels in *tau* KO and WT did not change post-SE. Quantification of western blot bands are shown in **C**. (**D**) Representative images of PLA for NR2B-PSD95 (cyan dots) co-labelled MAP2 (magenta) comparing WT versus *tau* KO demonstrated a reduction in SE-induced NR2B-PSD95 interactions in *tau* KO relative to WT post-SE. (**E**) Quantification of PLA puncta (complexes). (**F**) Representative immunoblots of hippocampal PSD-enriched fractions show that SAR treatment reduced SE-induced pSFK, pTau (AT8) and pNR2B levels, but not nNOS and pTau (Y18). The total Fyn, PSD95 and NR2B levels did not change between groups. (**G**) Quantification of western blots. Uncropped blots are included in Supplementary Fig. 6. Scale bar 100 µm. Bar graphs contain all data points and expressed as mean ± SEM. Dots represent individual animals. One-way ANOVA with Tukey’s *post hoc*.

To further determine whether strategies disrupting Fyn-tau interactions yield protection against the upregulation of Fyn-tau related signalling molecules in the hippocampus, we compared the changes between *tau* KO mice and WT mice challenged with KA. The experimental design is illustrated in Fig. 7A. Tau KO mice had little to no Fyn-tau puncta as in negative controls (Supplementary Fig. 5C). In the hippocampal PSD fraction, *tau* KO significantly reduced SE-induced pSFK-Y416 (*P* = 0.024), pNR2B (Y1472) (*P* = 0.0008) and nNOS (*P* = 0.0042) compared to WT mice at 24 h post-SE (Fig. 7B and C). The total Fyn, PSD95 and NR2B levels were unchanged between groups, and tau was completely absent in *tau* KO mice, validating the model. Moreover, PLA revealed a significant decrease in NR2B-PSD95 interactions (*P* = 0.0002) in *tau* KO relative to WT (Fig. 7D and E) following KA treatment, demonstrating the Fyn-tau interaction-dependent NR2B-PSD95 downstream signalling post-SE which is disabled in *tau* KO.

Since gene KO approach has a limited clinical application, we sought to determine whether pharmacological inhibition of Fyn/SFK activation disrupts Fyn-tau interactions and rescue SE-induced upregulation of the key phosphorylated PSD protein levels. The experimental design is illustrated in Fig. 7A. Synaptosomal PSD fractional analysis of the hippocampus revealed significant reduction of pSFK-Y416 (*P* = 0.021), pTau (AT8) (*P* = 0.035) and pNR2B (Y1472) (*P* = 0.043) levels in SAR-treated group relative to the VEH-treated group (Fig. 7F and G). SAR treatment, however, had no significant effects on SE-induced upregulation of nNOS (*P* = 0.1273) and Y18 levels (*P* = 0.9965) in PSD fraction (Fig. 7F and G).

### Saracatinib treatment after the onset of spontaneously recurring seizure, reduced Fyn-tau and NR2B-PSD95 interactions and spontaneously recurring seizure in rat chronic epilepsy

**Figure 8:**
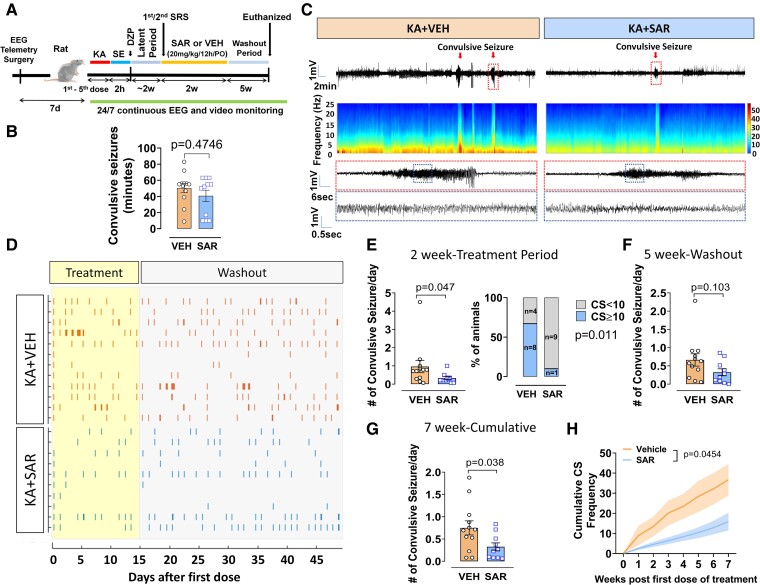
**SAR treatment for 2 weeks after the onset of SRS reduced recurrent convulsive seizures in the rat chronic epilepsy model.** (**A**) Experimental design of KA-induced chronic epilepsy and treated which SAR or VEH after the onset of one to two spontaneous convulsive seizures following *status epilepticus* (SE). (**B**) Initial SE severity comparison between animals in pre-assigned groups for SAR or VEH. (**C**) Representative EEG traces and multi-tapered spectrograms from VEH- or SAR-treated animals. Red arrows indicate the events of spontaneous convulsive seizures in both groups. Insets show magnified EEG traces (red- and blue-dashed boxes) from both groups. (**D**) Raster plot showing all SRS during the SAR or VEH treatment period and after (washout period): SAR (*n* = 10, blue bars) or VEH (*n* = 12, orange bars). (**E**) SAR treatment significantly reduced SRS/day during the 2-week SAR treatment. Bar graphs showing the percentage of animals with equal or greater than 10 SRS in SAR (1/10) and VEH (8/12) groups during the treatment period. Fisher’s exact test. (**F**) Average number of SRS per day during the washout period showed no difference between the groups. (**G**) Average number of SRS per day in a 7-week period showed a reduction of SRS in the SAR group. (**H**) Cumulative plot of SRS in SAR and VEH groups showing a reduction of seizure by SAR. Repeated measures two-way ANOVA with Sidak’s *post hoc*. Bar graphs displayed all data points and expressed as mean ± SEM. Dots represent individual animals. The Student’s *t*-test or the Mann-Whitney test was used for two-group comparison.

The experimental design is illustrated in Fig. 8A. There was no significant difference in SE severity scores between animals assigned to SAR or VEH group, indicating unbiased and balanced SE severity between groups (Fig. 8B). Epileptiform spikes and SRS are illustrated in Fig. 8C. SAR treatment significantly reduced the average number of convulsive SRS per day (*P* = 0.0473) compared to the VEH-treated group during the 2-week treatment in epileptic animals (Fig. 8D and E). Moreover, the number of animals that had equal or greater than 10 SRS per day during the treatment period was significantly less (*P* = 0.0115) in the SAR group (1/10) than in the VEH group (8/12), indicating the anti-epileptic effects of Fyn/SFK inhibition (Fig. 8E). There was, however, no significant reduction (*P* = 0.1034) in SRS by SAR treatment, compared to VEH, during the 5-week washout period (Fig. 8F). Nevertheless, SAR significantly reduced seizure progression during the 7-week video-EEG observation period (*P* = 0.0385, *P* = 0.0454 for cumulative CS frequency) (Fig. 8G and H). However, the spike rate and the seizure duration were not significantly different between groups (Supplementary Fig. 3).

**Figure 9:**
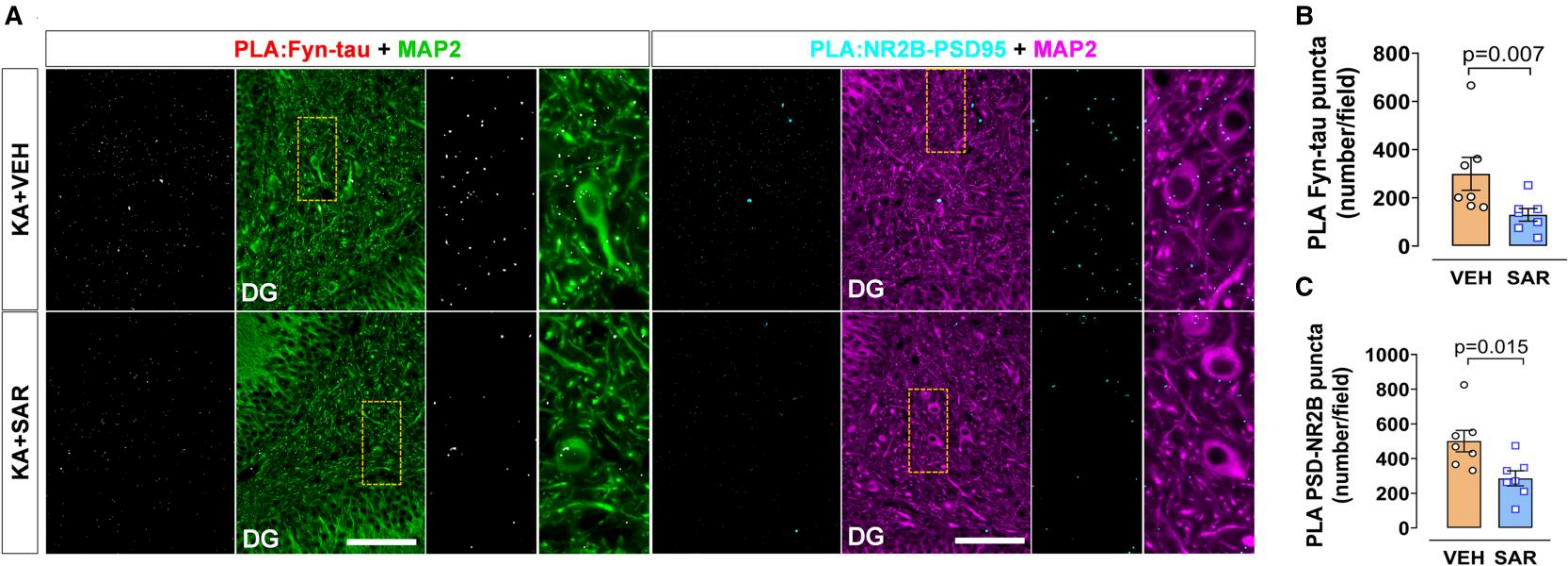
**SAR treatment for 2 weeks after the onset of SRS reduced the Fyn-tau and NR2B-PSD95 interactions in the rat chronic epilepsy model.** (**A**) Representative images of PLA for Fyn-tau (white dots) and NR2B-PSD95 (cyan dots) co-labelled with a dendritic marker MAP2 (green/magenta) comparing SAR versus VEH groups (**B, C**) demonstrating reduced Fyn-tau and NR2B-PSD95 interactions in SAR group. Scale bar 100 µm. Bar graphs displayed all data points and expressed as mean ± SEM. Dots represent individual animals. Two-group comparison used the Student’s *t*-test or the Mann–Whitney test.

PLA analysis of the hippocampus from these rats revealed a significant decrease of Fyn-tau (*P* = 0.007) and NR2B-PSD95 (*P* = 0.0385) complexes in the SAR group relative to the VEH group (Fig. 9A–C). Additionally, SAR treatment reduced epilepsy-induced upregulation of pSFK levels (*P* = 0.0265) but not pTau (AT8 and Y18) levels in the hippocampus (Supplementary Fig. 4). However, in the acute study (24 h post-SE), SAR-treated group showed significant reduction of AT8 (but not Y18) (Fig. 7F and G), suggesting a further long-term SAR treatment may be required to achieve therapeutic benefits.

## Discussion

Our present study provides evidence for the role of Fyn-tau and NR2B-PSD95 interactions in seizure-induced experimental models and in human epilepsy. Tau pathology has been associated with neuronal hyperexcitability and excitotoxicity in epilepsy models,^14,46-48^ human epilepsy^15-17,49-51^ and Alzheimer’s disease patients.^2^ On the other hand, Fyn promotes network excitability^6,7,24^ and seizure-induced neuroinflammation.^27,28^ The interactions between the SH3 domain of Fyn and PxxP_5/6_ motifs of tau contribute to neurotoxicity in Alzheimer’s disease.^20,33,34,52-54^. Understanding the significance of Fyn-tau interactions in seizures in epilepsy models may reveal therapeutic target/s for epilepsy.

Our novel data from the mouse and rat models of epilepsy showed a significant increase in phosphorylation of tau by Fyn/SFK. Moreover, SE caused hyperphosphorylation of tau at Ser202/Thr205 (AT8) at PSD, which is an early hyperphosphorylated site in Alzheimer’s disease,^55,56^ mediated by phosphorylation of CDK5 and GSK3β by Fyn.^57^ For Fyn to phosphorylate tau, Fyn must be in an active configuration.^52,58^ As shown in our previous studies^27,28^ and in this study, seizures increase Fyn activation (pSFK-Y416), which likely exposes its SH3 domains for tau interaction at PxxP_5/6_. Such interactions stabilize the enzymatic activity of Fyn.^20,21^ Interactions between Fyn and tau also mediate the localization of Fyn to the PSD to target NR2B.^34^ As reported by others,^59,60^ we also found that seizures enhance the phosphorylation of NR2B at Y1472 suggesting that Fyn-tau interaction may cause Fyn localization to PSD where it phosphorylates NR2B, which potentiates NMDAR function allowing Ca^2+^ and Na^+^ influx in neurons evoking aberrant neuronal excitation and seizures.^59^ Excessive calcium influx activates the calcium-dependent nNOS and persistent increase of the production of NO to cause neuronal death,^61,62^ which is also evident in our study. Furthermore, the increased number of Fyn-tau complexes in this study correlates with the increased number of NR2B-PSD95, evaluated by PLA and binding assays, suggesting that enhanced Fyn-tau interactions following SE may induce the formation of Fyn/tau/NR/PSD95 complexes to mediate seizure-induced aberrant network circuitry.

While previous studies have reported increased total Fyn and tau levels in the cytosolic and nuclear fractions following SE,^27,63^ the levels of postsynaptic total Fyn and tau surprisingly remain unchanged following SE in the present study, highlighting the importance of phosphorylation state of Fyn and tau, rather than their quantity, in facilitating seizure-induced neurotoxicity in the early phase of epileptogenesis. However, a study showed increased total Fyn expression in the PSD fractions in the presence of Aβ.^34^ We speculate that increased Fyn-tau interactions at the PSD following SE may not be necessarily caused by an increase in the total amount of Fyn and tau but more likely driven by the Fyn/SFK and tau phosphorylation and post-translational modifications could enhance their binding affinity.^52,58,64^ Although the pSFK antibody recognizes a conserved phospho-epitope at Y416 across all members of Src-family kinases, mounting evidence suggests the role of Fyn in facilitating hyperexcitability in epilepsy.^6,11,27,65^ Moreover, RNA-sequencing and gene ontology studies identified high expression of Fyn mRNA associated with hippocampal sclerosis in patients with epilepsy.^66^ Therefore, it is likely that increased phosphorylation of SFK in epilepsy is predominantly driven by the activation of Fyn.

**Figure 10:**
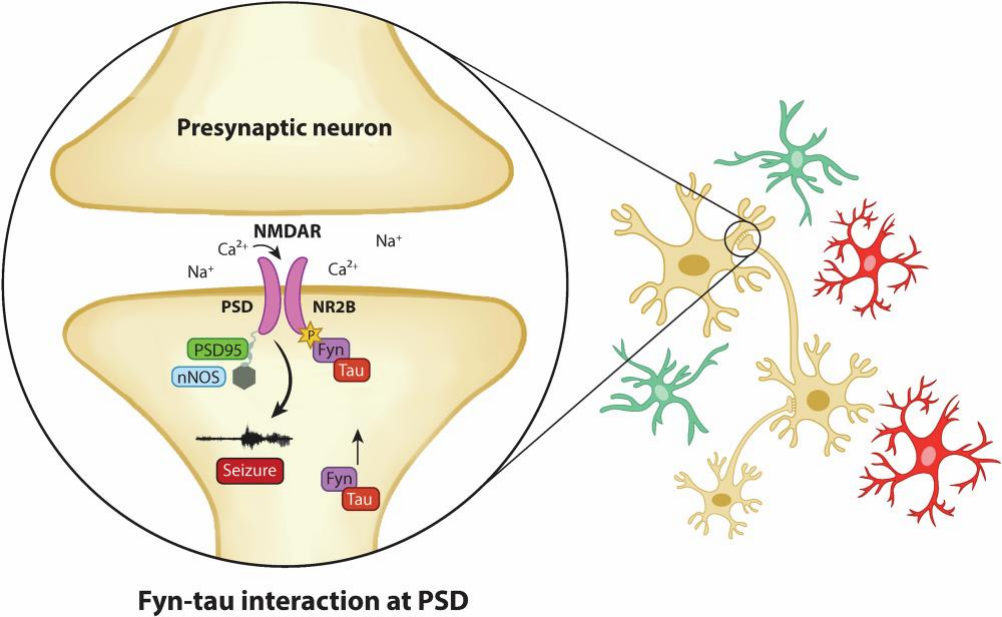
**Schematic illustration summarizing the mechanisms of the Fyn-tau signalling pathway in neurons.** Hyperphosphorylated tau serves as a chaperon to localize Fyn to the postsynaptic membrane, which further phosphorylates tau and stabilizes their interactions. Fyn then associates with PSD95 and phosphorylates NR2B at Tyr1472, facilitating their interactions. Upon activation of NR2B, calcium-dependent nNOS couples with PSD95 mediating the excessive Ca^2+^ and Na^+^ influx to cause persistent neuronal hyperexcitability (epileptiform spiking/seizures). These could, in turn, activate microglia and astrocytes to promote neuroinflammation and reduce seizure threshold leading to self-perpetuating seizures and neurodegeneration. Blue cells, neurons; green, astrocytes; red, microglia.

To further validate the Fyn-tau interactions identified by the PLA method, we performed binding assay, i.e. Co-IP on the hippocampal isolates from a rat model of epilepsy. In contrast to mouse model, KA-induced rat model of epilepsy mirrors human TLE with respect to SRS progression, neurodegeneration and gliosis.^67,68^ Fyn-tau association was enhanced 24 h following SE and increased the Co-IP of Fyn/tau/NR2B/nNOS/PSD95 complexes. The role of nNOS in pathogenesis of NMDAR-induced neurotoxicity has been extensively reported in many studies.^61,62,69^ The formation of these complexes mediates Aβ-induced excitotoxicity; thus, disruption of the interactions may have therapeutic benefit.^34,53,70^ Another study, however, reported that PSD95-nNOS interactions were not altered when NMDARs were chronically activated, despite the upregulated Fyn-tau interactions.^71^ We, therefore, hypothesize that the initial seizures that mediate the early formation of Fyn/tau/NR2B/PSD/nNOS complexes precede the overactivation of NMDAR in epilepsy (depicted in Fig. 10). This is supported by a recent discovery showing that Fyn-tau construct binds and engages PSD95 postsynaptically, resulting in abnormally clustered membrane-bound NMDAR which accounts for aberrant synaptic function.^72^ Another study showed that phosphorylation of Y18 in tau primarily facilitates NMDAR-dependent Ca^2+^ influx and neurotoxicity, but not through Fyn-tau binding.^32^ The latter study^32^ only examined the seventh PxxP motif of tau, which is not the primary binding target for Fyn.^52,73^ Therefore, it is likely that Y18 can be a potential mediator of SE-induced NMDAR toxicity in epilepsy models.

In the rat model of chronic acquired epilepsy, tau phosphorylation at AT8 and Y18 persisted in the hippocampus at 3 months post SE, consistent with increased tau pathology seen in KA-induced epileptic mice.^46^ Although phosphorylated tau promotes neuronal hyperexcitability,^6,8-10,32,74^ it is unclear whether tau pathology is a consequence of chronic seizures or a significant factor that drives the development of spontaneous seizures in epileptic brain. Moreover, tau pathology is often associated with cognitive decline in epilepsy;^15,16,49^ however, the role of tau phosphorylation at Y18 in epilepsy-induced cognitive deficit is unknown and needs further investigation. Nevertheless, our novel finding demonstrates the upregulation of pY18 in the chronic epilepsy model, perhaps as a consequence of enhanced Fyn-tau binding, and may serve as a marker for disease progression in the epileptic brain. Moreover, the increase of one of the AT8 phospho-epitopes at pSer202, but not pThr205, in the serum of epileptic animals could serve as a peripheral biomarker of neurodegeneration.^75^

The upregulated levels of Fyn-tau and PSD95-NR2B interactions were sustained in chronic epilepsy, and there was a strong correlation between these complexes and the frequency of SRS and epileptiform spikes, implying their role in seizures. However, we lack direct evidence to demonstrate whether enhanced coupling of Fyn-tau in chronic epilepsy aggravates NR2B-mediated currents to generate SRS and epileptiform spikes. Several studies demonstrated the presence of tau promoted hippocampal epileptiform discharge^7^ and altered NR2B-mediated currents,^7,32,35^ and these effects were exacerbated by Fyn.^7^ Therefore, reducing Fyn or tau or both disrupts the interactions and reduces seizure burden.^7,11,27^ In addition, Fyn-tau complexes showed a positive linear relationship with the levels of NR2B-PSD95 complexes, Fyn activation and tau phosphorylation at Y18, indicating their collective role in brain pathology. Therefore, our findings from this study and the literature on Alzheimer’s disease suggest the role of Fyn-tau interactions in brain pathogenesis in both Alzheimer’s disease and epilepsy.^19,76^ In the epilepsy model, the mechanism of brain pathogenesis may be predominantly driven by seizures, independent of Aβ, as the formation of these pathogenic Fyn/tau/NR2B/PSD95 complexes occurs in the early phase of epileptogenesis.^11^ However, a recent study in an experimental TLE model demonstrated that amyloidogenic pathways promote tau pathology during early epileptogenesis.^48^ Similarly, another group has shown the deposition of Aβ in the hippocampus of chronic human TLE,^16^ suggesting possible early interactions between Fyn-tau complexes and amyloid pathways during epileptogenesis that may persist in the chronic stage of epilepsy.

Several studies have demonstrated the presence of hyperphosphorylated tau and Aβ in the hippocampus and cortex that correlate with cognitive dysfunction in human epilepsy.^15-17,49,77^ In contrast, one study reported that tau pathology and Aβ are not as prevalent as reported in the literature and not necessarily correlate with cognitive impairments in epilepsy.^78^ In the present study, apart from the increased pTau (AT8), we, for the first time, also showed the upregulation of pY18 and Fyn/SFK activation (pSFK) levels accompanied by increased Fyn-tau interactions in human epilepsy. However, the limitation of our human study is that there is no information on seizure severity and cognitive dysfunction in these patients with epilepsy. Furthermore, we also acknowledge that our temporal lobe samples were limited to cortical epileptic foci and did not include the hippocampus. Furthermore, a representative Fyn-tau Co-IP also confirms the occurrence of such interactions in temporal cortex of an epileptic patient (Fig. 6K). We observed neuronal loss and gliosis in this cohort, consistent with the hallmarks of epilepsy in humans reported in the literature.^79,80^ In addition, NR2B-PSD95 interaction was markedly increased in the cortex of the human epileptic brain, which also complements previously reported studies.^81-83^ Similar to the findings in our animal models, the degree of Fyn-tau interactions positively correlated with NR2B-PSD95 complexes and tau phosphorylation, and activated Fyn levels in human epileptic brain. The binding of Fyn-tau also correlated with microgliosis suggesting its contribution to neuroinflammatory state in the human epileptic brain. Taken together, corroborating our animal studies, our human data highlight a pivotal role for Fyn/tau/NR2B/PSD95 interactions involving tau phosphorylation, Fyn/SFK and NMDAR activation in mediating seizures and disease-associated pathophysiological changes in epilepsy.

Since sustained Fyn-tau interactions facilitate neuronal hyperexcitability,^34,70^ we hypothesized that disrupting the interactions either by deleting tau or Fyn or pharmacological inhibitors will dampen hyperexcitability and validate the role of Fyn-tau interactions in an animal model of epilepsy. Many, including our publications, have shown that tau ablation is protective against seizures^7-11^ and Fyn/SFK inhibition modifies the progression of epilepsy.^7,25,27^ Our KO mouse study, indeed, revealed that depletion of tau prevented SE-induced hyperexcitability, perhaps by inactivating Fyn and NR2B and thereby reducing the formation of NR2B-PSD95 complexes. In transgenic *fyn* KO (Tg/*fyn*^−/−^) mice where tauopathy mice crossed with *fyn* KO mice, there was near-complete ablation of neurofibrillary tangles, reduced tau hyperphosphorylation, altered tau solubility and diminished synaptic tau accumulation.^57,84^ Gene KO approach is not clinically feasible; therefore, we inactivated Fyn/SFK using a pharmacological inhibitor SAR in the rat model, which suppressed seizure-induced postsynaptic activation of NR2B and tau phosphorylation at AT8, but not Y18 and nNOS. SAR treatment also reduced spontaneous seizures during the 2-week treatment when the drug was administered after the onset of the first or second spontaneous seizures. It is likely that Fyn/SFK inhibition protects neurons by targeting Fyn-tau interactions rather than directly reducing the level of pY18 or nNOS, a similar finding was also reported by others.^54^ It is also likely that tau phosphorylation at Y18 is regulated by other kinases independent of Fyn/SFK that are also affected by seizures as the Y18 remains phosphorylated in spite of the absence of Fyn in transgenic Alzheimer’s disease models.^57,84^ Alternatively, SAR dosing optimization may be required to reverse the early upregulation of tau phosphorylation (Y18) in epilepsy.

In summary, our study demonstrates the role of Fyn-tau interactions in mediating seizure-induced brain pathology in animal models of both early stage of epileptogenesis and chronic epilepsy through recruitment of PSD95/NR2B/nNOS interactions (depicted in Fig. 10). The results also validated the occurrence of these interactions in human epileptic brains. Therefore, given the importance of Fyn-tau interactions in epilepsy, presented in this study, a therapeutic strategy to prevent their interactions could protect against seizures by targeting PSD95-NR2B/nNOS interactions^34,85^ and neurotoxicity.^86^ Other studies also demonstrated the protective effects in stroke and mild TBI models when PSD95-nNOS interactions were disrupted.^61,62^ Recently, tat-Tau PxxP_5/6_, a peptide targeting Fyn-tau interactions, prevented neurotoxicity induced by Aβ *in vitro*, while the efficacy of the peptide has not been tested in *in vivo.*^53,87^ Blocking Fyn and tau interactions with this peptide inhibitor could further validate the outcomes of pharmacological inhibition of Fyn/SFK by SAR in epilepsy model and identifies Fyn-tau interaction as a potential therapeutic target in epilepsy.

## Supplementary Material

fcae327_Supplementary_Data

## Data Availability

The data that support the findings of this study are available from the corresponding author upon request.
