# Supplementary material for: Enhanced Fyn-tau and NR2B-PSD95 interactions in epileptic foci in experimental models and human epilepsy
Source: Brain Commun. 2024 Sep 19;6(5):fcae327. doi: 10.1093/braincomms/fcae327 (PMC11444080; doi:10.1093/braincomms/fcae327)
Supplement: fcae327_Supplementary_Data [file fcae327_supplementary_data.pdf]

Supplementary Materials and Methods

Image processing

Images containing 10 z-stacks at 1.5 μm steps were obtained and compressed into a maximum contrast projection with the "EBImage" package (available at https://github.com/aoles/EBImage and https://github.com/DragonDuck/MaxContrastProjection) on R studio version 1.11.463 (Bioconductor, MA, USA) software before analysis. For the mouse/rat brain sections, 10 z-stacked images of the hippocampus taken at 20x were sampled for analysis.

Synaptosomal and post-synaptic density samples preparation

Subcellular fractionation was carried out with some modifications from previously described studies[1–3]. Hippocampal and cortical tissues were first homogenized in ice-cold sucrose buffer (0.32 M sucrose, 10mM HEPES, 1% protease-phosphatase inhibitor (PPI, pH 7.4, Thermo). The lysates were centrifuged at 1000x *g* at 4^0C for 10 mins generating the supernatant (S1) containing total protein (TP) and the nuclear-enriched pellets (P1). TP supernatants were collected for further analysis and centrifuged at 12,000xg for 20min at 4^0C to obtain cytosolic supernatant (S2) and crude synaptosomal fraction enriched pellets (P2). While S2 was stored at -80^0C for later use, pellets (P2) enriched in crude synaptosomal fraction were resuspended in washed buffer (4mM HEPES, 1mM EDTA, pH 7.4) and centrifuged at 12,000x g for 20 mins at 4^0C for two cycles. The supernatants were discarded and the pellets were resuspended in buffer A (20 mM HEPES, 100 mM NaCl, 0.5% Triton X-100, 1% PPI, pH 7.2) for 15 mins on a rotator followed by centrifugation at 12,000x g for 20 mins at 4^0C. The supernatant (S3) of this process had soluble non-PSD fractions rich in extrasynaptic proteins. The pellets (P3) were then resuspended in buffer B (20 mM HEPES, 0.15 mM NaCl, 1% Triton X-100, 0.1% SDS, 1% deoxycholate, 1% NP-40, 1% PPI, pH 7.5) for 1 h on a rotator at 4^0C and later centrifuged at 10,000x g for 15 mins. The resultant supernatants (S4) contain detergent-insoluble PSD-enriched fractions (Supplementary Figure 6A). All fractions were aliquoted and stored at -80 ^{0}C. The abundance of proteins was evaluated by Western blotting with specific primary antibodies (Supplementary Figure 6B).

ELISA

Phosphorylated tau (Ser202 and Thr205) levels in the sera were determined using ELISA kits (LS Bios, GA, USA). The procedure followed was per the manufacturer's instructions with minor modifications appropriate for serum samples. Briefly, 50 µL of coating buffer (R&D systems, MN, USA) was added to a 96-well plate and incubated overnight at room temperature. The plate was washed 3 times with 1x wash buffer and blocked with blocking buffer for 1h at room temperature. After washes, serum was diluted in coating buffer (1:4 dilution) and added to the wells and incubated overnight at room temperature to enable the phosphorylated tau proteins to bind the wells of the plate to serve as antigens. The plate was then washed and incubated with 50 uL of either pTau-Ser202 or pTau-Thr205 primary antibodies, overnight at 4^0C. In some wells, total tau antibody was also included for normalization of pTau levels. Following washes, the plate was incubated with 50uL of HRP-conjugated secondary antibodies for 1.5 hours at room temperature. Ready-to-use substrate reagent was added and incubated for 30 mins at room temperature, followed by the addition of stop solution containing H_2SO_4. Immediately after the contents of the wells turned golden yellow, the plate was read for the protein abundance at OD 450 nm using SpectraMax M2 Gemini Molecular Device Microplate reader (Molecular Device, PA, USA). The levels of pTau in serum were expressed as relative to the total Tau levels against the relative absorbance values at OD 450 nm. All assays were performed in triplicate.

References

1. Liu G, Thangavel R, Rysted J, et al. Loss of tau and Fyn reduces compensatory effects of MAP2 for tau and reveals a Fyn-independent effect of tau on calcium. *Journal of Neuroscience Research*. 2019;97(11):1393-1413. doi:10.1002/jnr.24517

2. Milnerwood AJ, Gladding CM, Pouladi MA, et al. Early Increase in Extrasynaptic NMDA Receptor Signaling and Expression Contributes to Phenotype Onset in Huntington's Disease Mice. *Neuron*. 2010;65(2):178-190. doi:10.1016/j.neuron.2010.01.008

3. Li C, Götz J. Somatodendritic accumulation of Tau in Alzheimer's disease is promoted by Fyn-mediated local protein translation. *The EMBO Journal*. Published online September 1, 2017:e201797724. doi:10.15252/embj.201797724

Supplementary Table 1 Clinical information and tissue availability of human control, epilepsy, AD cases

CASE	GROUP	AGE (YEARS)	SEX	PMI (HOURS)	COD	BRAIN SECTIONS AVAILABLE	BRAAK STAGE	USE
1	Control	11	Male	17	Infection	Temp CTX, Hipp	n/a	IHC
2	Control	36	Female	14	Metastatic Carcinoma	Temp CTX, Hipp	n/a	IHC
3	Control	40	Male	18	Valve replacement	Temp CTX, Hipp	n/a	IHC
4	Control	54	Female	24	Severe Obesity	Temp CTX, Hipp	n/a	IHC
5	Control	37	Female	19	Bilateral PE	Temp CTX, Hipp	n/a	IHC
6	Epilepsy	24	Male	n/a	n/a	Lateral Temp CTX	n/a	IHC
7	Epilepsy	49	Female	n/a	n/a	Lateral Temp CTX	n/a	IHC
8	Epilepsy	30	Male	n/a	n/a	Lateral Temp CTX	n/a	IHC
9	Epilepsy	37	Male	n/a	n/a	Right frontal CTX	n/a	IHC
10	Epilepsy	38	Male	n/a	n/a	Right frontal CTX	n/a	IHC
11	Epilepsy	51	Male	n/a	n/a	Lateral Temp CTX	n/a	IHC
12	AD	72	Male	20	n/a	Lateral Temp CTX	III, ABC Score (A3, B3, C2)	IHC
13	Control	63	Male	22	ILD	Temp CTX, Hipp	n/a	IP, WB
14	Epilepsy	35	Male	n/a	n/a	Temp CTX, Hipp	n/a	IP, WB

Abbreviations: AD: Alzheimer's Disease, PE: Pulmonary Embolism, ILD: Interstitial Lung Disease, PMI: Postmortem Interval, Temp: Temporal, CTX: Cortex, Hipp: Hippocampus, IHC: Immunohistochemistry, WB: Western Blotting, IP: Immunoprecipitation, n/a: Not Applicable.

Supplementary Table 2 Antibodies and reagents used in the experiments.

Antibody Name	Host	Clonality	Suppliers	Working Dilution	Catalog Number	Use
			**Primary antibodies**			
Fyn	Mouse	Monoclonal	ThermoFisher	1:1000	MA1-19331	WB
Fyn	Rabbit	Polyclonal	Sigma-Aldrich	1:500 (WB), 1:200 (IHC, PLA)	HPA023887	WB, IHC, PLA
Total Tau (DA9)	Mouse	Monoclonal	Peter Davies	1:50	RRID: AB_2716723	PLA, IHC
Total Tau (Tau5)	Mouse	Monoclonal	ThermoFisher	1:1000	AHB0042	WB
Total Tau (DD8)	Rabbit	Polyclonal	Gloria Lee	1:1000		WB
NR2B	Mouse	Monoclonal	Santa-Cruz	1:50 (PLA), 1:1000 (WB)	sc-365597	PLA, WB
NR2B	Rabbit	Polyclonal	Abcam	1:1000 (WB)	ab65783	WB
PSD-95	Rabbit	Polyclonal	Invitrogen	1:1000	51-6900	WB
Synapsin Ia/b	Mouse	Monoclonal	Santa-Cruz	1:1000	sc-390867	WB
a-tubulin	Mouse	Monoclonal	Santa-Cruz	1:5000	sc-5286	WB
nNOS	Sheep	Polyclonal	PC Emson	1:5000	RRID: AB-2314957	WB
MAP-2	Chicken	Polyclonal	ThermoFisher	1:500	PA1-10005	IHC
IBA1	Goat	Polyclonal	Abcam	1:300	ab5076	IHC
GFAP	Mouse	Monoclonal	Sigma-Aldrich	1:400	AB5804	IHC
NeuN	Rabbit	Polyclonal	Sigma-Aldrich	1;200	MAB377	IHC
pSFK (Y416)	Rabbit	Monoclonal	Cell Signaling	1:1000	2101	WB
pSFK(Y419)	Rabbit	Polyclonal	Invitrogen	1:100	44-660G	IHC
pTau (AT8)	Mouse	Monoclonal	ThermoFisher	1:1000, (WB), 1:100 (IHC)	MN1020	WB
pTau (Y18)	Mouse	Monoclonal	Gloria Lee	1:1000, (WB), 1:100 (IHC)		WB, IHC
pTau (Y18)	Mouse	Monoclonal	Medimabs	1:1000, (WB), 1:100 (IHC)	MM-0194-P	WB, IHC
pNR2B (Y1472)	Rabbit	Polyclonal	Phospho Solutions	1:500	p1516-1472	WB
B-Actin	Mouse, Rabbit	Monoclonal	Sigma-Aldrich	1:10000	A5316	WB
**Primary antibodies for Immunoprecipitation**						
Total Tau (DA9)	Mouse	Monoclonal	Peter Davies	1;50	RRID: AB_2716723	IP
PSD-95	Mouse	Monoclonal	Sigma-Aldrich	4 μg	MAB1598	IP
IgG Isotype control (IgG2a)	Rat	Monoclonal	BioLegend	4 μg	407102	IP
IgG Isotype control (IgG1)	Rat	Monoclonal	BioLegend	4 μg	406602	IP
Secondary antibodies						
Biotin-SP-AffiniPure Donkey	Goat, mouse, rabbit		Jackson IR	1:300		IHC
Streptavidin Rhodamin X, Alexa Flour 488, AMCA Blue			Jackson IR	1:300		IHC
Rhodamin X, Alexa Flour 488-AffiniPure Donkey Anti Rabbit IgG (H+L)	Chicken Mouse, Rabbit		Jackson IR	1:300		IHC
IRDye® 800CW or 680LT Donkey IgG (H + L)	Mouse, Rabbit, Sheep		LI-COR Biosciences	1:10000	926-32213	WB
IRDye® 680RD Detection Reagent			LI-COR Biosciences	1:10000	926-69100	WB

Reagents or Drugs	Source	Identifier
(-)-(a)-Kainic Acid (hydrate)	Cayman Chemicals	78050
Diazepam USP Injection	Hospira Inc	
Baytril (enrofloxacin)	Bayer	
4,6-diamidino-2- phenylindole (DAPI)	Vector Laboratories	H-1200-10
Duolink Insitu PLA probe anti-rabbit PLUS	Sigma-Aldrich	DUO92002
Duolink Insitu PLA probe anti-mouse MINUS	Sigma-Aldrich	DUO92004
Duolink Insitu Detection Reagent Orange	Sigma-Aldrich	DUO92007
Protein A/G Magnetic Beads	ThermoFisher	88802
ELISA kit tau (Ser519/202)	LifeSpan Bioscie	LS-F1644
ELISA kit tau (Thr205)	LifeSpan Bioscie	LS-F1670
ELISA Plate-coating Buffer	R&D Systems	DY006
Donkey Normal Serum	Abcam	ab7475

Supplementary Table 3 Details of statistical analysis applied for each experiment/figure.

FIGURE	PANEL		TEST
1	C	AT8	Unpaired t-test, two-tailed t=3.957, df=10; p=0.0027
		Y18	Unpaired t-test, two-tailed t=4.144, df=10; p=0.0020
	E	pNR2B	Unpaired t-test, two-tailed t=3.057, df=10; p=0.0121
		NR2B	Unpaired t-test, two-tailed t=0.8077, df=10; p=0.4381
		pTau (AT8)	Unpaired t-test, two-tailed t=5.165, df=10; p=0.0004
		pTau (Y18)	Mann-Whitney, two-tailed, p=0.0022
		Total Tau	Unpaired t-test, two-tailed t=0.1978, df=10; p=0.8472
		pSFK	Unpaired t-test, two-tailed t=2.363, df=10; p=0.0398
		Fyn	Unpaired t-test, two-tailed t=1.232, df=10; p=0.2460
		nNOS	Unpaired t-test, two-tailed t=3.765, df=10; p=0.0037
		PSD-95	Unpaired t-test, two-tailed t=0.3155, df=10; p=0.7589
2	B	Fyn-tau	Unpaired t-test, two-tailed t=5.682, df=10; p=0.0002
		NR-PSD	Unpaired t-test, two-tailed t=4.039, df=10; p=0.0024
	C	Fyntau vs NRPSD	Spearman Correlation, R= 0.7832, 95%CI= 0.3636 to 0.9386, p= 0.0038
	E	Fyn	Unpaired t-test, two-tailed t=5.741, df=8; p= 0.0004
		Tau	Unpaired t-test, two-tailed t=0.6333, df=8; p= 0.5442
	G	NR2B	Unpaired t-test, two-tailed t=4.301, df=8; p=0.0026
		Fyn	Unpaired t-test, two-tailed t=4.458, df=8; p=0.0021
		nNOS	Mann-Whitney, two-tailed, p=0.0317
		Tau	Unpaired t-test, two-tailed t=2.862, df=8; p=0.0211
		PSD95	Unpaired t-test, two-tailed t=1.433, df=8; p=0.1898
3	D	NeuN	Unpaired t-test, two-tailed t=4.606, df=5; p=0.0058
		GFAP	Unpaired t-test, two-tailed t=4.488, df=5; p=0.0065
		IBA1	Unpaired t-test, two-tailed t=4.637, df=5; p=0.0056
4	C	pTau (AT8)	RM-two-way ANOVA: F (1, 13) = 8.201; P=0.0133; Sidak's post-hoc
		pTau (Y18)	RM-two-way ANOVA: F (1, 13) = 18.09; P=0.0009; Sidak's post-hoc
	E	pSFK	Unpaired t-test, two-tailed t=2.704, df=14; p=0.0171
	F	pSer202	Unpaired t-test, two-tailed t=3.114, df=10; p=0.0110
		pThr205	Unpaired t-test, two-tailed t=1.983, df=10; p=0.0755
		Total Tau	Unpaired t-test, two-tailed t=1.272, df=10; p=0.2322
5	C	Fyn-tau	RM-two-way ANOVA: F (1, 13) = 11.62; p=0.0047; Sidak's post-hoc;
		NR-PSD	RM-two-way ANOVA: F (1, 13) = 10.24 p=0.0070; Sidak's post-hoc;
	D	Fyntau vs Seizure/day	Spearman Correlation, R= 0.7697, p= 0.0126
		Fyntau vs Spike/day	Spearman Correlation, R= 0.6970, p= 0.0306
		Fyntau vs Duration	Spearman Correlation, R= -0.06667, p= 0.8651
	E	NRPSD vs Seizure/day	Spearman Correlation, R= 0.7212, p= 0.0234
		NRPSD vs Spike/day	Spearman Correlation, R= 0.6970, p= 0.0544
		NRPSD vs Duration	Spearman Correlation, R= 0.1515, p= 0.6821
	F	Heatmap rat	Correlational Matrix, Spearman correlation
6	B	NeuN	Unpaired t-test, two-tailed t=2.198, df=9; p= 0.0555
		IBA1	Mann-Whitney, two-tailed, p= 0.0087
		GFAP	Unpaired t-test, two-tailed t=2.746, df=9; p= 0.0226
	D	pTau(AT8)	Mann-Whitney, two-tailed, p= 0.0043

	F	pTau(Y18)	Unpaired t-test, two-tailed t=2.589, df=9, df=10; p=0.0292
	H	pSFK	Unpaired t-test, two-tailed t=5.767, df=9, df=10; p=0.0003
	J	Fyn-tau	Mann-Whitney, two-tailed, p= 0.0303
		NR-PSD	Unpaired t-test, two-tailed, t=2.931, df=9; p= 0.0167
	L	Heatmap human	Correlational Matrix, Spearman correlation
7	C	pNR2B	one-way ANOVA: F (3, 15) = 11.98; p=0.0003; Tukey's post-hoc;
		NR2B	one-way ANOVA: F (3, 15) = 0.03095; p=0.9924; Tukey's post-hoc;
		nNOS	one-way ANOVA: F (3, 15) = 11.30; p=0.0004; Tukey's post-hoc;
		PSD95	one-way ANOVA: F (3, 15) = 0.6123; p=0.6175; Tukey's post-hoc;
		pSFK	one-way ANOVA: F (3, 15) = 5.580; p=0.009; Tukey's post-hoc;
		Fyn	one-way ANOVA: F (3, 15) = 0.6634; p=0.5873; Tukey's post-hoc;
	E	NR-PSD	one-way ANOVA: F (3, 18) = 18.25; p<0.0001; Tukey's post-hoc;
	G	pNR2B	one-way ANOVA: F (2, 12) = 8.369; p=0.0053; Tukey's post-hoc;
		NR2B	one-way ANOVA: F (2, 12) = 2.245; p=0.1485; Tukey's post-hoc;
		nNOS	one-way ANOVA: F (2, 12) = 5.474; p=0.0204; Tukey's post-hoc;
		PSD-95	one-way ANOVA: F (2, 12) = 2.748; p=0.1041; Tukey's post-hoc;
		pTau (AT8)	one-way ANOVA: F (2, 12) = 5.641; p=0.0187; Tukey's post-hoc;
		pTau (Y18)	one-way ANOVA: F (2, 12) = 7.733; p=0.0070; Tukey's post-hoc;
		Total Tau	one-way ANOVA: F (2, 12) = 1.033; p=0.3855; Tukey's post-hoc;
		pSFK	one-way ANOVA: F (2, 12) = 10.46; p=0.0023; Tukey's post-hoc;
		Fyn	one-way ANOVA: F (2, 12) = 1.680; p=0.2274; Tukey's post-hoc;
8	B	SE Severity	Mann-Whitney, two-tailed, p= 0.4746
	E	2 week	Mann-Whitney, two-tailed, p= 0.0473
		% seizures-2 week	Fisher's exact test, two-sided, p=0.0115
	F	5 week	Mann-Whitney, two-tailed, p= 0.1034
		7 week	Unpaired t-test, two-tailed t=2.7215, df=20; p=0.0385
	H	7 week-Cum. CS	RM-two-way ANOVA: F (1, 20) = 4.553; P=0.0454; Sidak's post-hoc;
9	B	Fyn-tau	Mann-Whitney, two-tailed, p= 0.0070
	C	NR-PSD	Unpaired t-test, two-tailed t=2.831, df=12; p=0.0385
S1	B	pNR2B	Unpaired t-test, two-tailed t=4.114, df=10; p=0.0021
		NR2B	Unpaired t-test, two-tailed t=0.02050, df=10; p=0.9840
		pTau(AT8)	Unpaired t-test, two-tailed t=4.603, df=10; p=0.0010
		pTau(Y18)	Unpaired t-test, two-tailed t=2.796, df=10; p=0.0189
		Total Tau	Unpaired t-test, two-tailed t=0.6131, df=10; p=0.5535
		pSFK	Unpaired t-test, two-tailed t=2.863, df=10; p=0.0169
		Fyn	Unpaired t-test, two-tailed t=0.3151, df=10; p=0.7592
		nNOS	Unpaired t-test, two-tailed t=2.305, df=10; p=0.0439
		PSD-95	Unpaired t-test, two-tailed t=0.1450, df=10; p= 0.8876
S2	B	Tau	Unpaired t-test, two-tailed t=1.600, df=9; p= 0.1440
		Fyn	Unpaired t-test, two-tailed t=1.208, df=9; p= 0.2578
S3	A		RM-two-way ANOVA: F (1, 21) = 0.7217; P=0.7908; Sidak's post-hoc;
	B	2 week	Mann-Whitney, two-tailed, p= 0.6744
	C	5 week	Unpaired t-test, two-tailed t=0.4488, df=20; p=0.6584
	D	7 week	Unpaired t-test, two-tailed t=0.5267, df=20; p=0.5267
	E	2 week	Mann-Whitney, two-tailed, p= 0.1229
	F	5 week	Unpaired t-test, two-tailed t=1.602, df=19; p=0.1255

	G	7 week	Unpaired t-test, two-tailed t=1.684, df=20; p=0.1076
S4	D	pSFK	Unpaired t-test, two-tailed t=2.715, df=8; p= 0.0265
		pTau(AT8)	Unpaired t-test, two-tailed t=1.507, df=12; p=0.1576
		pTau(Y18)	Unpaired t-test, two-tailed t=0.9249, df=12; p=0.3733

Supplementary Figures

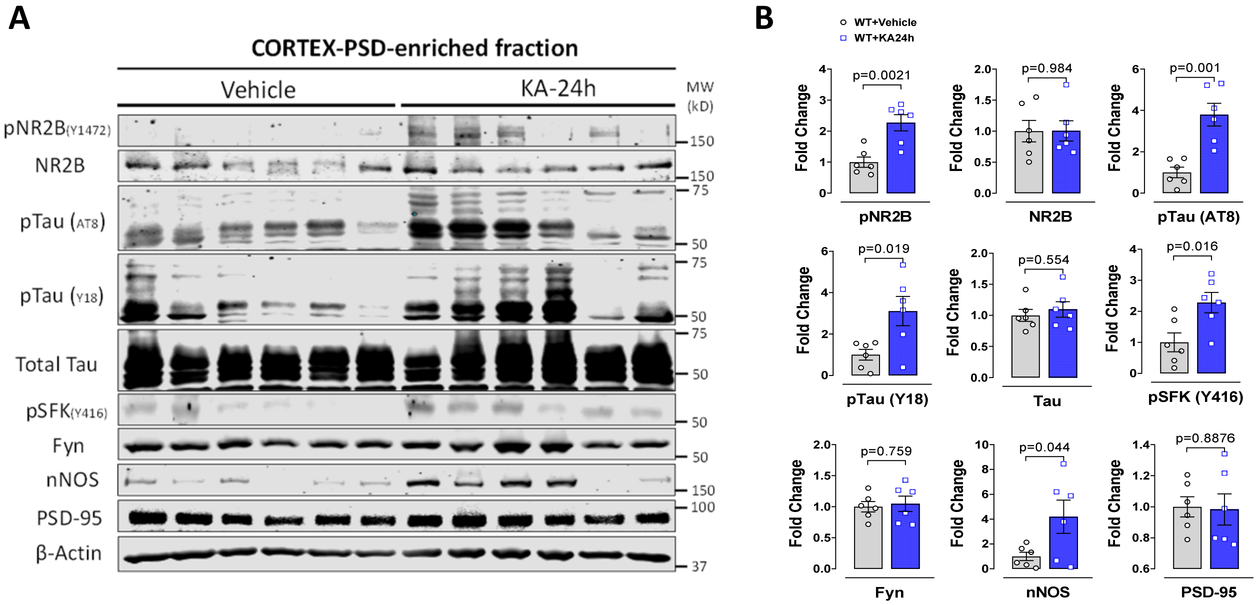

Supplementary Figure 1. Elevated hyperphosphorylation of tau, activation of Fyn, NMDA receptors in mouse cortex following SE at 24h. (A) Representative immunoblots of PSD-enriched fraction of cortex comparing vehicle vs. KA treated mice show increased phosphorylation of (B) NMDAR (pNR2B), tau (AT8, Y18), Fyn activation (pSFK), and levels of nNOS similar to that of in the hippocampus. The levels of Fyn, total Tau, PSD-95 are unaltered following KA. Data displayed all data points and expressed as mean±SEM. Two-group comparison used Student's t-test or Mann-Whitney test.

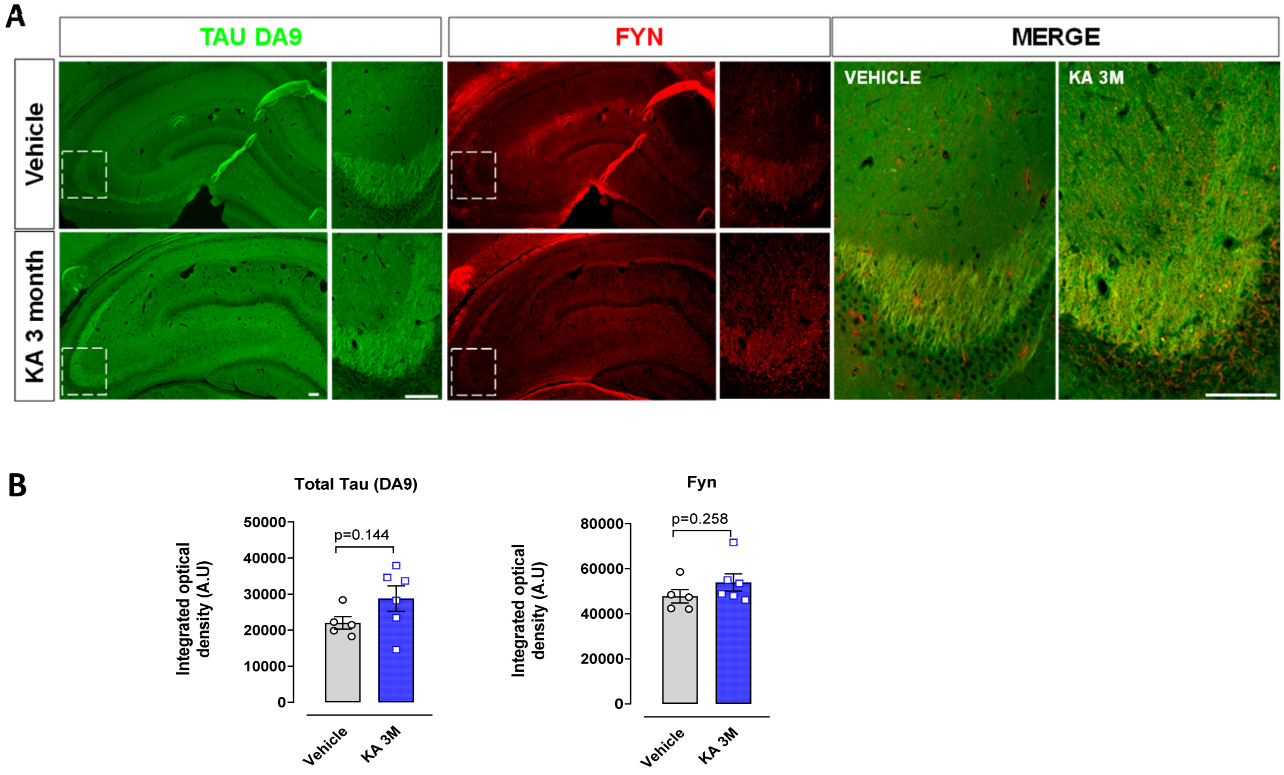

Supplementary Figure 2. **Impacts of Chronic epilepsy on Fyn-tau levels in the hippocampus**. (**A**) Representative immunostaining of tau (DA9) coupled with Fyn in the hippocampus show unchanged levels of (**B**) Fyn and Tau in the KA treated rats relative to control rats. (A.U. Arbitrary Unit). Scale bar 100 µm; for magnified images, scale bar 50 µm. Bar graphs displayed all data points and expressed as mean±SEM. Two-group comparison used Student's t-test or Mann-Whitney test.

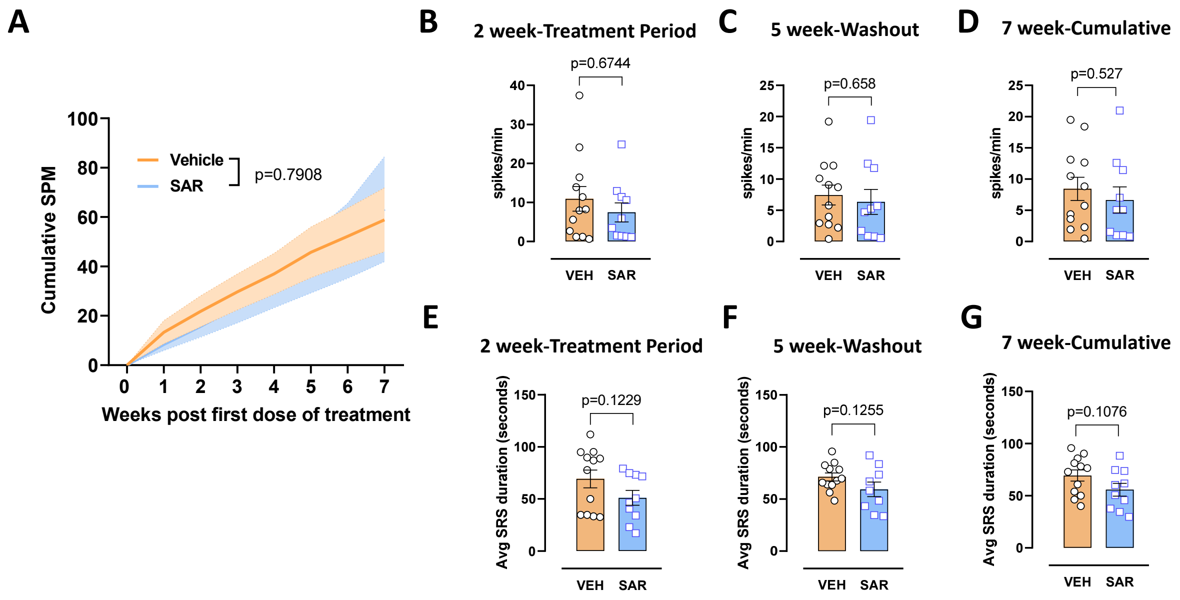

Supplementary Figure 3. **The effects of Fyn inhibition on epileptiform spikes and duration of seizures**. (**A**) Cumulative plot of spikes/min comparing SAR and VEH treatment groups. Repeated Measures-two-way ANOVA with Sidak's post-hoc. (**B**) Average number of spikes/min comparing SAR ad VEH groups during 2-week treatment period, (**C**) 5 week-washout period, (**D**) total 7 week-cumulative period. (**E**)) Average duration of spontaneous convulsive seizure comparing SAR ad VEH groups during 2-week treatment period, (**F**) 5 week-washout period, (**G**) total 7 week-cumulative period. Bar graphs displayed all data points and expressed as mean±SEM. Two-group comparison used Student's t-test or Mann-Whitney test.

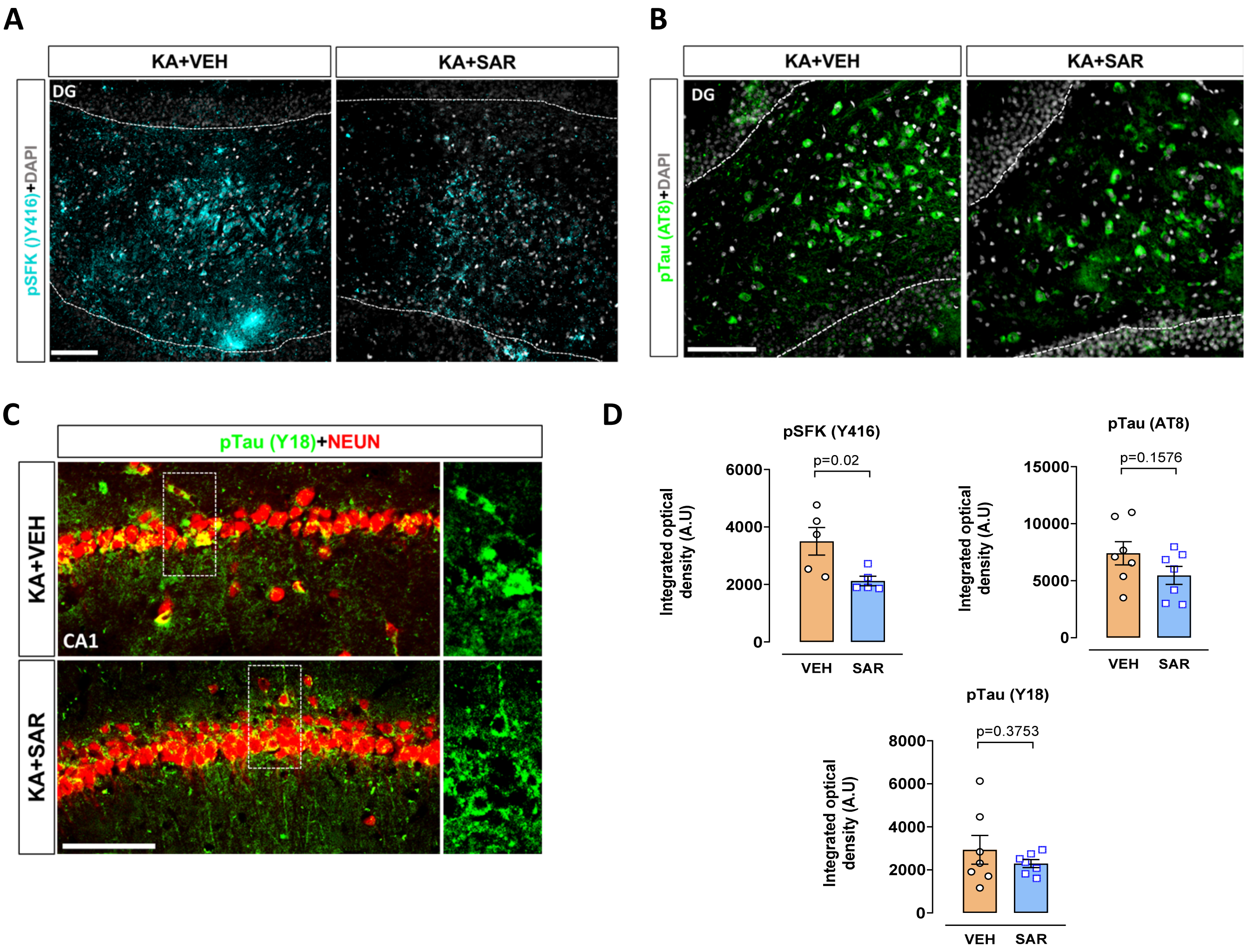

Supplementary Figure 4. **The effects of Fyn inhibition on epilepsy-induced activated Fyn and tau hyperphosphorylation**. (**A**) Representative immunostaining of activated Fyn marker (cyan) with pSFK (Y416), (**B**) phosphorylated Tau at AT8 (green) in both groups. (**C**) Representative images of co-immunostaining between phosphorylated Tau epitope at Y18 (green) and a neuronal marker (Neun, red) comparing Vehicle and SAR groups following KA Injection. Magnified areas (Dashed box) showing extent of Y18 staining in both groups. (**D**) Quantification of fluorescence staining intensity in A, B, and C. relative to Vehicle rats. (A.U. Arbitrary Unit). Scale bar 100 µm. Bar graphs displayed all data points and expressed as mean±SEM. Two-group comparison used Student's t-test or Mann-Whitney test.

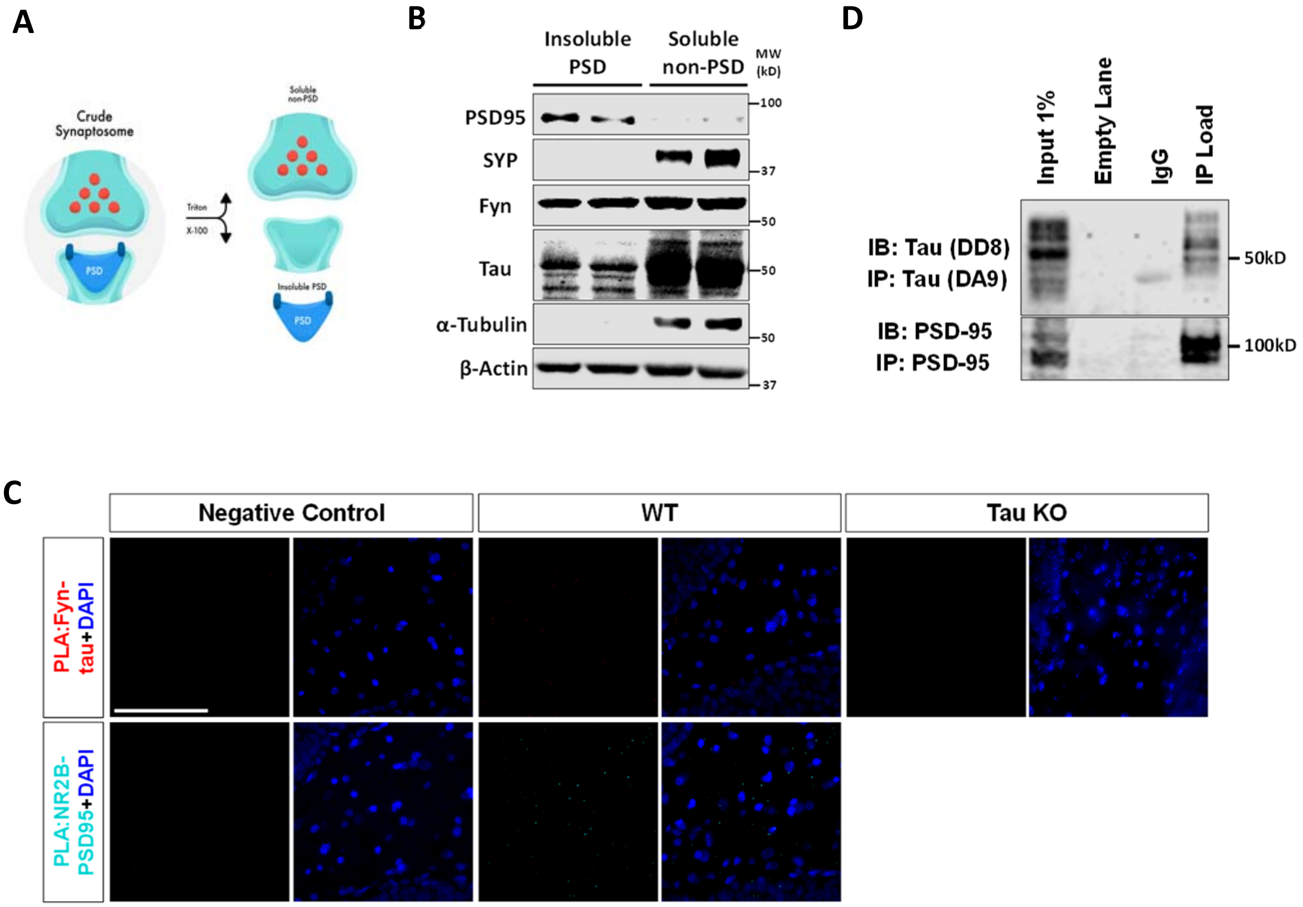

Supplementary Figure 5. **Validation of subcellular fractionation and Proximity Ligation Assays for Fyn-tau-NR2B-PSD95 complexes.** (**A**) Schematic of PSD fractionation from Crude synaptosome (adapted from Liu et al. (5772001090717) with permission (**B**) Representative immunoblots show enrichment of synaptophysin (SYP), a presynaptic marker, and GAPDH in non-PSD fraction whereas enrichment of PSD95, a postsynaptic marker, is observed in insoluble PSD fraction. Fyn and tau are present in Insoluble PSD (PSD-enriched fraction) and soluble Non-PSD fraction. (**C**) Representative images of PLA for Fyn-tau (red dots) NR2B-PSD95 (cyan dots) including negative control, in which one of the primary antibodies is omitted, WT and tau KO. Scale bar 100 µm. (**D**) Representative Immunoblots of immunoprecipitation of tau (DA) and PSD-95 from hippocampal lysates of control brains showing enrichment of tau and PSD-95 in IP load whereas IgG lane shows no enrichment of tau and PSD95.

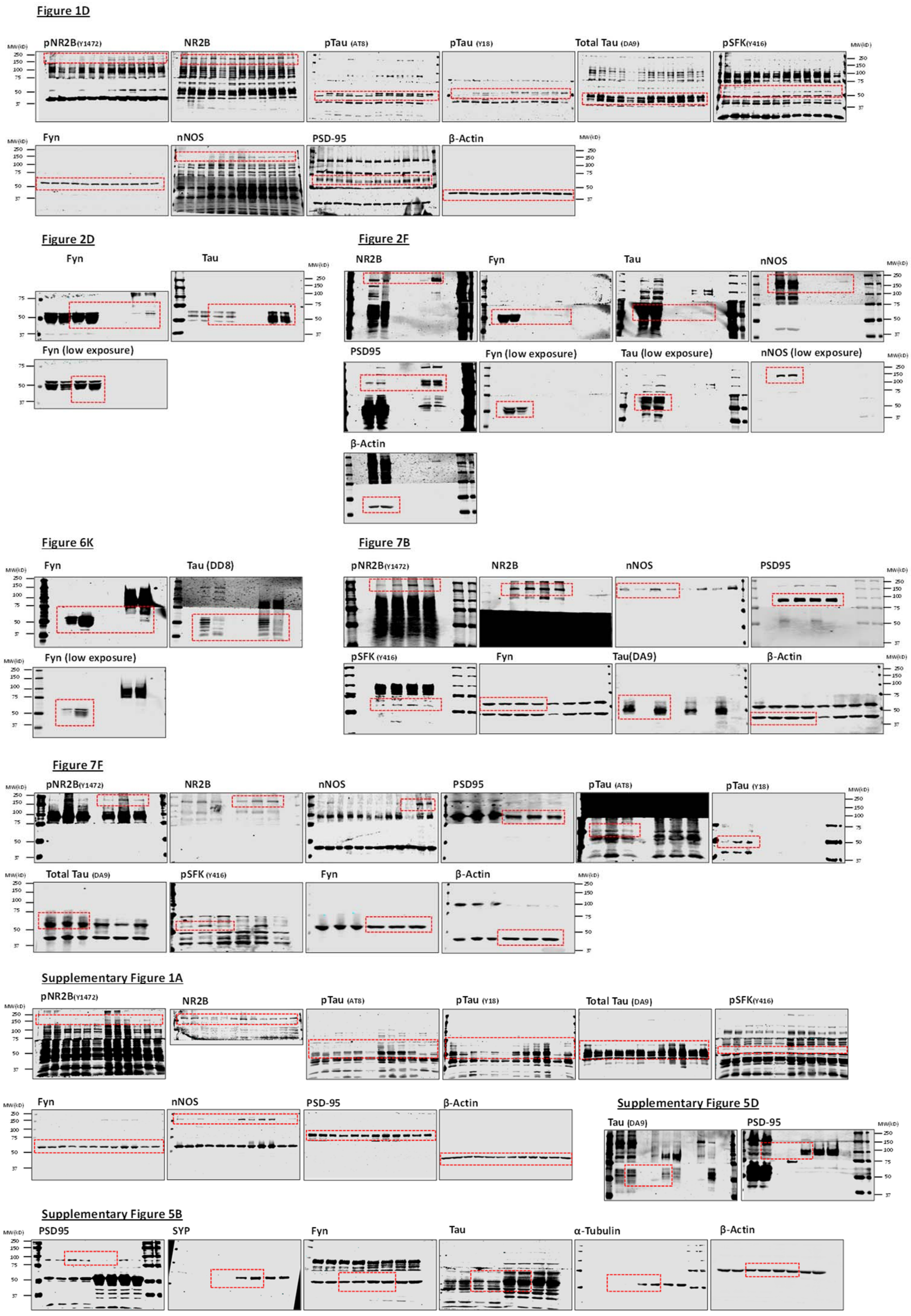

Supplementary Figure 6 Full membranes detected for Western blots shown in (red) indicated main and supplementary figures. Red boxes highlight the sections of blots shown in indicated figures.
